# Identification of a small molecule targeting EPLIN as a novel strategy for the treatment of pediatric neuroblastoma and medulloblastoma

**DOI:** 10.1038/s41419-025-07876-7

**Published:** 2025-07-23

**Authors:** Emma Lindell, Jing Guo, Miao Zhao, Natallia Rameika, Xi Lu, Tabea Wacker, Lei Zhong, Tobias Bergström, Sara Svanberg, Azazul I. Chowdhury, Peter Bergsten, Xingqi Chen, Daniel Bexell, Fredrik J. Swartling, Tobias Sjöblom, Xiaonan Zhang

**Affiliations:** 1 Department of Immunology, Genetics and Pathology, Uppsala University, SE-751 85 Uppsala, Sweden; 2 Centre for Computational Biology, Duke-NUS Medical School, 8 College Road, Singapore, 169857 Singapore; 3 Department of Medical Cell Biology, Uppsala University, BMC, Husargatan 3, SE-75123 Uppsala, Sweden; 4 Division of Translational Cancer Research, Department of Laboratory Medicine, Lund University, Lund, 22381 Sweden

**Keywords:** Paediatric cancer, Target identification

## Abstract

Amplification of the MYCN proto-oncogene serves as a key marker of aggressive disease and poor treatment outcomes in certain pediatric tumors originating from the nervous system, including neuroblastoma and medulloblastoma. However, the complex nature of the challenging MYCN protein underscores the urgent need for additional targets and therapies to tackle neuroblastoma and medulloblastoma. In this study, with a primary focus on neuroblastoma and the aim of also benefiting children with medulloblastoma, we identified FLIX5, a small compound that exhibits broad cytotoxicity against both neuroblastoma and medulloblastoma cells, primarily by triggering apoptosis. Furthermore, FLIX5 enhances the cholesterol dependency of neuroblastoma cells under conditions where mitochondrial function is impaired. FLIX5 as well shows a synergistic effect when combined with vincristine, a conventional anticancer drug, against neuroblastoma cells and organoids. Through proteome integral solubility alteration, computational molecular docking predictions, and cellular thermal shift assays for target identification and validation, FLIX5 reveals EPLIN (Epithelial Protein Lost In Neoplasm) as a previously unexplored drug target. EPLIN is involved in several cellular processes, including cholesterol uptake and mitochondrial function. The discovery of FLIX5 targeting EPLIN presents new opportunities for treating malignant pediatric tumors, with the potential to target chemoresistant dormant cancer cells and broaden its therapeutic applications to other tumor types.

## Introduction

Neuroblastoma stands as the most prevalent solid tumors in children, exhibiting a wide spectrum of clinical behaviors [1]. The amplification of the MYCN proto-oncogene emerges as a crucial driver correlating with aggressive disease and poor treatment outcomes. Particularly, metastatic MYCN-driven tumors represent a significant challenge with survival rates below 50% [2, 3]. Medulloblastoma, similar to neuroblastoma as a common malignant tumor in children, frequently involves MYCN amplification and is associated with a poor prognosis [4]. However, given the challenges associated with targeting the MYCN protein, there remains an urgent need to identify novel therapeutic targets that act independently of MYCN in the treatment of these pediatric tumors.

In cell culture models, spheroids more closely mimic the properties of tumors than monolayer cultures with regard to growth kinetics and metabolic rates [5, 6]. Moreover, tumor cells are in general more resistant to anticancer agents when the cells are grown as spheroids rather than monolayer cultures [7, 8], reflecting the acquired resistance at the multicellular level.

This study, with a primary focus on neuroblastoma and the aim of also benefiting children with medulloblastoma, we screened a library of 1581 chemical compounds using both monolayer and spheroid models. Through our efforts, we identified FLIX5, a small molecule that exhibits broad cytotoxicity against both neuroblastoma and chemoresistant medulloblastoma cells, primarily by inducing apoptosis. Importantly, FLIX5 demonstrated a significant therapeutic window, showing selectivity between normal and cancer cells. Through the target identification and validation of FLIX5, we discovered EPLIN (Epithelial Protein Lost In Neoplasm) as a previously unexplored drug target. EPLIN is a key regulator of cytoskeletal dynamics, cell motility, growth, and metabolism [9]. Our findings may offer a novel approach for treating high-risk neuroblastoma, while also benefiting children with medulloblastoma, with the potential for broader applications to other tumor types.

## Methods and materials

### Chemicals and antibodies

FLIX5 was obtained from the DTP developmental therapeutics program. Vincristine, paclitaxel, docetaxel, and vinorelbine were obtained from plates of NCI approved oncology drug set (The NCI Diversity Set VII). Staurosporine (Cat. #HY-15141) was obtained from MedChemExpress (NJ, USA). Etoposide (Cat. #S1225) from Selleckchem (Germany). The primary antibodies anti-mouse Actin (Cat. #sc-47778) was retrieved from Santa Cruz Biotechnologies (Dallas, TX, USA), anti-mouse-MYCN (Cat. #16898) from Abcam (Cambridge, UK), and anti-rabbit-EPLIN (LIMA1) (Cat. #HPA052645) and anti-rabbit-SRRD (Cat. #NBP1-70715) from Sigma-Aldrich (St Louis). The antibodies anti-rabbit Cleaved PARP (Cat. # #5625), anti-rabbit Cleaved Caspase-3 (Cat. #9661S), rabbit-anti-ɣH2AX (Cat. #2577), rabbit-anti p-4EBP1 (Cat. #2855), rabbit-anti LC3A/B (Cat. #12741) and rabbit-anti p53 (Cat. #9282) were obtained from Cell Signaling Technology (Danvers, MA, USA).

### Cell culture

hTERT-immortalized retinal pigment epithelial cell line (Cat. #CRL-4000) and neuroblastoma cell lines SK-N-AS (Cat. #CRL-2137), SH-SY5Y (Cat. #CRL-2266), SK-N-SH, (Cat. #HTB-11), CHP-212 (Cat. #CRL-2137), IMR-32 (Cat. #CRL-127), SK-N-BE2 (Cat. #CRL-2271) and medulloblastoma cell lines HDFa (Cat. #PCS-201-012), D283 (Cat. #HTB-185) and Daoy (Cat. #HTB-186) was obtained from (ATCC, Manassas, VA, USA). hTERT, SK-N-AS, SH-SY5Y, SK-N-SH, CHP-212, IMR-32, SK-N-BE [2] was maintained in 1:1 ratio of EMEM (ATCC, Cat. #30-2003) and Ham’s F-12 Nutrient Mix (Thermo Fisher Scientific, Waltham, MA, USA, Cat. #11765054) supplemented with 1% penicillin streptomycin (Thermo Fisher Scientific, Cat. #15140-122) and 10% FBS (Thermo Fisher Scientific, Cat. #10270-106). Daoy was maintained in EMEM supplemented with 1% penicillin streptomycin and 10% FBS. HDFa and D283 was maintained in DMEM + Glutmax (Thermo Fisher Scientific, Cat. #61965026) supplemented with 1% penicillin streptomycin and 10% FBS. GMYC1 [10], GTML-S1, GTML2 and GTML3 [11] were maintained in Neurobasal—A medium (Thermo fisher scientific, Cat. #10888022) supplemented with B27-A (Thermo Fisher Scientific, Cat. #12587010), 1% penicillin streptomycin, 1% L-glutamine (100x) (Thermo fisher Scientific, Cat. #25030081), 20 ng/mL bFGF (Thermo Fisher Scientific, Cat. #100-18B), 20 ng/ml EGF (Sigma Aldrich, Cat. #E9644). All cells were maintained at 37 °C and 5% CO_2_. All cell lines were authenticated by STR profiling (ATCC cell authentication service) and the MycoAlert mycoplasma detection kit (Lonza) was used to frequently test for mycoplasma infection.

### Spheroid generation

Spheroids was generated by plating 10,000 cells/well in 100 µL medium in 96-well ultra-low attachment plates (Corning, NY, USA, Cat. #7007). Cells were settled in the wells for 20 min followed by centrifugation at 1000RPM for 5 min and cultured in 37 °C in 5% CO_2_. Spheroids were cultured for 4 days before drug exposure and images was taken by Incucyte SX5 (Sartorius, Germany) followed by calculation of the spheroids volume by: V = (4/3*π) *r^3^

### Drug screening and hit confirmation

The NCI Diversity Set VII containing 1581 compounds was derived from the almost 140,000 compounds available for distribution from the DTP repository (compound list), was initially screened at 10 μM concentration using 96-well plates at a single dose on CHP-212 cells. This screening process was repeated three times, resulting in the identification of 484 compounds that exhibited toxicity (causing more than 20% cell death after 72 h of treatment). These 484 compounds were then evaluated in SK-N-BE2 spheroids, known for their resistance to chemotherapies, at a concentration of 10 μM. After this step, 125 compounds remained, as they induced more than 20% cell death in the spheroids. Subsequently, the concentration of the remaining 125 compounds was reduced to 1 μM, and they were tested on five different spheroid models (SK-N-BE2, IMR-32, CHP-212, SH-SY5Y and SK-N-AS) for 72 h. Among these, 37 compounds exhibited toxicity exceeding 20% at 1 μM concentration and were retained for further evaluation. To further narrow down the selection of compounds, the 37 identified compounds were retested on the aforementioned spheroid models. Only compounds showing more than 50% cell death under each spheroid model were considered. Following this criterion, five compounds were ultimately retained for further investigation.

### Cell viability assay

A resazurin based cell viability assay was performed to analyze the compounds toxicity. To assess the cell viability cells were seeded in a 96-well plate. After overnight incubation cells were exposed to indicated treatment for 72 h followed by incubation with resazurin for 2–4 h. Spheroids were exposed to respective compound for 72 h after spheroids were generated, followed by incubation with resazurin overnight. Measurement of the fluorescent intensity was made by the CLARIOstar microplate reader at excitation (545–20 nm) and emission at (600–40 nm). The fluorescent background from all values was subtracted and the cell viability was calculated as follow, (fluorescence intensity of drug exposed cells—fluorescence intensity of background)/(fluorescence intensity of DMSO exposed control—fluorescence intensity of background) ×100. Using the nonlinear regression, [Inhibitor] vs. normalized response analysis, in GraphPad prism 9, the half-maximal inhibitory concentration (IC50) was calculated.

### Colony formation assay and crystal violet staining

SK-N-AS spheroids exposed to FLIX5 for 72 h at indicated concentrations were washed in 100 µL HBSS and dissociated in 100 µL trypsin at 37 °C and further detached by consequent pipetting. The cell suspension was transferred to a 24-well plate containing 2 mL of culture medium and cultured until the cells under control conditions became confluent. Cells were fixated with 600 µL ice cold methanol for 5 min at room temperature. After removal of the methanol, cells were stained with 600 µL crystal violet staining solution (0.5 g crystal violet powder in 80 mL H_2_O and 20 mL methanol) for 30 min at room temperature. Following the staining, cells were washed, 3*H_2_O, and left to dry at room temperature. Using ImageJ [12], the staining intensity was determined by running the commands Lab Stack and measurement of the mean gray value. The fold change was calculated for each condition compared to control.

### Proteome integral solubility alteration (PISA) for target identification

The proteome integral solubility alteration (PISA) assay is a technique that provides detailed, quantitative analysis of changes in the soluble fraction of proteins in response to drug treatment. Deep proteomic analysis is then performed to compare drug-treated samples with controls [13]. For target identification, PISA was conducted in CHP-212 cells following a 60-min treatment with FLIX5 (70 nM), and compared to a control group treated with DMSO: DMSO (*n* = 4), FLIX5 (*n* = 3). For each experiment, equal amounts of peptides resulting from different samples will be labeled using TMTpro 16-plex technology and one final multiplex sample will be obtained from each experiment. The last will be fractionated off-line by microscale preparative reverse chromatography into fractions to be run on LC-MS. LC-MS of all fractions will be conducted using Nano-LC-ESI_MS/MS on a high resolution Orbitrap Exploris480. Proteome Discoverer was used for database search and quantification against the Uniprot Homo sapiens (Human) protein database UP000005640. Datasets were normalized on total intensity and normalization on average of controls and relative *p* values (student’s *t* test) for statistical significance of variation. Proteome discoverer search with quantification analysis of the spectra generated by LC-MS/MS of fractionated sample was able to identify and quantify the following:

**Table:** 

Peptide sequences ID (identified with TMT labeling)	194,573
Protein ID after contaminants removal across all replicates	9744
Protein ID and quantified with ≥2 unique peptides across all replicates	8497

Score value was calculated as: (-Log10 p value * Log2 fold change) in PISA result (Supplementary Table 1).

### Computational molecular-docking analysis for target validation

#### Structure prediction

AlphaFold algorithm was employed for predicting protein structure prediction. The default AlphaFold model parameters were employed for the predictions. These parameters include the use of multiple sequence alignments (MSAs) derived from the input protein sequence, as well as the consideration of predicted contact maps and other features. Predicted structures were prepared using SwissPDB viewer and visualized using Discovery Studio visualizer.

#### Protein-ligand docking

For initial blind (unbiased) docking, the grid box was defined to encompass the whole protein. For targeted docking, grid box was defined to encompass the binding site of the protein, predicted from the blind docking method. AutoDock-vina algorithm was employed for all docking calculations. AutoDock Vina employs an empirical scoring function to evaluate the binding affinity of ligands. The Lamarckian Genetic Algorithm was used to explore the ligand conformational space, and multiple docking runs were performed to improve the reliability of the results. The ligand flexibility was considered during docking. Torsional bonds in the ligand were allowed to rotate to explore different conformations, enhancing the chances of finding the optimal binding pose. The predicted binding affinities for each docking pose were extracted from the AutoDock Vina output. These values were used to rank and prioritize the potential binding poses.

#### Molecular dynamic simulations

The protein-ligand complexes were initially prepared for molecular dynamic simulations. The complexes were solvated in a triclinic box filled with Simple Point Charge (SPC) water molecules, and NaCl ions were added to maintain a physiological salt concentration (0.15 M NaCl). The solvated system underwent energy minimization using the steepest descent method for 5000 steps to eliminate steric clashes and optimize the geometry. Subsequently, the system was equilibrated for 300 ps with positional restraints on the protein and ligand to facilitate proper solvent equilibration. Molecular dynamics simulations were conducted either for 1 ns, for initial Gibbs binding free energy estimation, or for 500 ns using the GROMACS simulation package. The resulting molecular dynamics trajectories were analyzed using tools available in GROMACS. Key parameters such as root mean square deviation (RMSD) and the number of protein-ligand hydrogen bonds were monitored to ensure system stability during the simulation. Snapshots were extracted from the trajectory at regular intervals for subsequent Molecular Mechanics Poisson-Boltzmann Surface Area (MMPBSA) calculations.

#### MMPBSA calculations

The Molecular Mechanics Poisson-Boltzmann Surface Area (MMPBSA) method was employed to estimate the binding free energy of the protein-ligand complex. Snapshots from the molecular dynamics trajectory were used to perform separate energy calculations for the complex, the isolated ligand, and the isolated protein using the Generalized Born (GB) and Poisson-Boltzmann (PB) continuum solvent models. The binding free energy (ΔG_bind) was calculated as ΔG_bind = ΔE_MM - TS + ΔG_solvent, where ΔE_MM is the difference in molecular mechanics energy, TS is the entropic contribution, and ΔG_solvent is the solvation free energy. The binding free energy obtained from MMPBSA was further processed to calculate the Gibbs free energy using the relationship ΔG = −RT ln(Keq), where R is the gas constant and T is the temperature. Statistical analysis, such as error estimation and confidence intervals, was performed to evaluate the reliability of the calculated binding free energies.

### Thermal shift assay for target confirmation

Procedures primarily followed the protocol described in [14]. In brief, CHP-212 cells were cultured in T-75 flasks until they reached 80-90% confluency and exposed to DMSO control or 0.5 µM FLIX5 for 1 h, with 2 flasks per condition. The cells were collected, washed with HBSS, and resuspended in HBSS with 1:100 protease inhibitor (Sigma-Aldrich, St. Louis, MO, USA, Cat. #P8340). 100 µL of the cell suspension was distributed into PCR tubes and kept on ice. Using a PCR thermal cycler, the cells were subjected to a heating treatment starting at 40 °C with a 3 °C increment up to 67 °C for 3 min, followed by cooling on ice. Cells were lysed by three freeze-thaw cycles in liquid nitrogen, followed by centrifugation at 13,300 RPM for 20 min at 4 °C. The supernatant was mixed with half the volume of SDS-PAGE reducing loading mix. 20 µL was loaded on 1.5 mm NuPAGE 12% Bis-Tris mini protein gels and run with 20X MOPS buffer (Invitrogen, Cat. #NP0001), followed by detection of EPLIN (1:1000) and SRRD (1:1000).

### Immunostaining of spheroids

IMR-32 spheroids were generated as previously described. After 7 days incubation at 37 °C the spheroids were fixated in 4 mL of 4% formalin overnight and then dehydrated for 2 h in 70% EtOH. The spheroids were then embedded in 200 µL of HistoGel (Epredia, UK, Cat. #HG-4000-012) and transferred to cassettes (Simport Scientific, Canada, Cat. #M490-2), for paraffin embedding. The paraffin embedded spheroids were cut in 5 mm sections using a microtome, and incubated overnight at 37 °C. The slides were deparaffinized and rehydrated by sequential immersion for 5 min, two times each, in Xylene, 100% EtOH, 90%EtOH, 70%EtOH and one time in deionized H_2_O. Antigen retrieval was performed by immersing the slides in a 1:100 dilution of Antigen Unmasking solution (Vector laboratories Inc, CA, USA, Cat. #H-3300) and microwaved for 15 min at 100 W. The slides were washed 3times 4 min in TBST buffer (Sigma-Aldrich, St. Louis, MO, USA, Cat. #91414-100TAB) followed by blocking with 2.5% Normal Horse Serum (Vector Laboratories Inc, Cat. #S-2012), in a humidity chamber for 25 min. The slides were incubated overnight, in humidity chamber, with primary antibodies anti-mouse-ki67 (1:200) and anti-mouse-p27 (1:200), diluted in 2.5% Normal Horse Serum. Following incubation, slides were washed 3times 4 min in TBST and incubated for 15 min with ImPRESS (Peroxidase) Polymer Anti-Mouse IgG reagent (Vector Laboratories Inc, Cat. #MP-7402) in the humidity chamber. The slides were washed 3times 4 min in TBST and developed using DAB Substrate Kit (Vector Laboratories Inc, Cat. #SK-4100), following protocol for 5 to 20 min. Lastly, the slides were washed 3times 4 min in TBST and for 4 min in deionized H_2_O before mounting.

### Western blots

Cells were seeded at 60% confluency and exposed to 2*IC50 of FLIX5 for 24 or 48 h. The cells were lysed with 1:100 RIPA buffer (Thermo Fisher Scientific, Cat. #89901) containing protease and phosphatase inhibitors (Sigma-Aldrich, St. Louis, MO, USA, Cat. #PPC1010). Protein concentration was measured using BCA assay (Thermo Scientific, Cat. #23227). Samples were run on 1.5 mm NuPAGE 12% Bis-Tris mini protein gels (Invitrogen, Cat. #NP0355BOX) according to the protocol. Proteins were transferred to iBlot™ 2 Transfer Stacks, nitrocellulose membrane (Invitrogen, Cat. #IB23001) using the iBlot® 7-min blotting system (Invitrogen, Cat. #IB21001). Membranes were blocked in 5% milk-PBST for 1 h and then probed with primary antibodies against Actin (1:1000), MYCN (1:1000), Cleaved PARP (1:1000), Cleaved Caspase-3 (1:500), p53 (1:1000), SRRD (1:1000), EPLIN (1:1000), ɣH2AX (1:1000), p-4EBP1 (1:1000) and LC3A/B (1:1000) overnight in 5% BSA-PBST. The following day, membranes were washed with PBST and incubated with the corresponding secondary antibodies, anti-mouse (1:5000, Invitrogen, Cat. #31430) or anti-rabbit (1:5000, Invitrogen, Cat. #31460), in 5% milk-PBST for 1 h. The signal was detected using SuperSignal™ West Pico PLUS Chemiluminescent Substrate (1:1, Thermo Scientific, Cat. #34577) with an Amersham Imager 680 (GE Healthcare, Chicago, IL, USA).

### Generation of EPLIN KO cells using CRISPR cas9

IMR-32 and DAOY EPLIN KO cell pool was generated by Synthego (CA, USA) using the sgRNA sequence:

CCAUCAUACCUUCAUUGCUUCUGGACUUAGUGGCUUGGGAAACUCAAGCAGGUGAC

UCCC. PCR forward primer TCAGTGTGCTCTTCTGTGGC and revers primer GGACCAGAGAATACCTGAGCC. To generate clones, single cells were seeded into individual wells of a 96-well plate. The expanded single-cell clones were then transferred to 6-well plates and cultured until confluence. Two clones (KO1 and KO2), were selected for downstream experiments.

### Bioinformatics analyses

The data analysis using the public dataset E-MTAB-10301. Raw fastq datasets were downloaded from data source. All samples passed quality check (i.e., sequencing quality, number of reads aligned). Reads were mapped to human reference GRCh38 and mouse reference GRCm38 accordingly by STAR aligner (version 2.7.11b). Counts were quantified by featureCounts (version 2.0.6). Differential expression tests were performed by DESeq2 version 1.38.3. The data consist of three groups: mouse_ko, mouse_oe, human_oe (Supplementary Table 2). Differential expression analysis on human OE vs Control, mouse OE vs Control, mouse KO vs WT were performed (Supplementary Table 2, *mm.hs.sig.anno.xlsx* file has all the differential expressed genes, significance p.adj <0.05 in either KO or OE group) that have consistent results from OE and KO groups (up in OE and down in KO, and verse vasa). The human differential expression test results are listed for reference and not used as criteria. Using the gene list in the table, the over-representation tests and gene set enrichment analysis (GSEA) were performed for pathway enrichment using Reactome, Gene Ontology Biological Process, Hallmark using R package clusterProfiler version 4.6.2.

Publicly available datasets were obtained from and analyzed using the R2: Genomics Analysis and Visualization Platform (http://r2.amc.nl). The SEQC498 (ag44kcwolf), Asgharzadeh-249, Cangelosi-786, and Bell-97 datasets, which include 498, 249, 786, and 97 neuroblastoma samples, respectively, were used for Kaplan-Meier analysis based on EPLIN gene expression. The correlation between EPLIN expression and overall survival rate in both neuroblastoma datasets was compared using the first_vs_last_quartile cutoff mode, and statistical significance was calculated using the log-rank test. EPLIN expression in cell lines was obtained from the Human Protein Atlas (https://www.proteinatlas.org).

### Mitochondrial activity assay using Seahorse XF analyzer

The mitochondrial effect in cells exposed to FLIX5 was analyzed using the seahorse cell mito-stress test kit (Agilent Technologies, Cat. #103015-100) with a Seahorse XF analyzer. For this experiment, 40,000 CHP-212 or SK-N-BE2 cells per well were plated in an XF96 well plate and allowed to attach at 37 °C for at least 6 h. Following attachment, the cells were treated overnight with 50 nM FLIX5. The next day, the medium was replaced with Seahorse assay medium, and the plates were incubated without CO2 at 37 °C for 1 h. The assay protocol was as follows: measure the basal oxygen consumption rate (OCR) levels three times; Inject 1.5 μM oligomycin through port A and measure OCR levels three times; inject 1 μM FCCP through port B and measure OCR levels three times and inject 0.5 μM rotenone and 0.5 μM antimycin through port C and measure OCR levels three times. To calculate ATP-related respiration OCR, the average OCR values post-oligomycin injection were subtracted from the basal OCR values. For calculating the maximum mitochondrial respiration capacity, the average OCR values post-rotenone/antimycin injection were subtracted from the averaged maximal OCR values obtained post-FCCP injection.

### ATP assay

CHP-212 and Daoy cells were seeded in a 96-well white surface plates (Thermo Fisher, Cat.136101). After overnight incubation CHP-212 cells were exposed to FLIX5 for 24 and 48 h. The ATP was measured by 1:1 Cell Titer Glo and the luminescence was read using CLARIOstar microplate reader and normalized to replicate resazurin based cell viability assay.

### Free and total cholesterol measurement

To analyze the free and total cholesterol the Cholesterol/Cholesterol Ester-Glo^TM^ Asssay (Promega, WI, USA, Cat. #J3190) kit was used. Briefly, 12,000 CHP-212 cells were seeded in 96-well white surface plates (Thermo Fisher, Cat. #136101) and incubated overnight. The cells were then exposed to FLIX5 for 48 h. Following exposure, the cells were washed twice with 100 µL HBSS and lysed with 40 µL cholesterol lysis solution. The plates were shaken and incubated for 30 min at 37 °C. Subsequently, 40 µL of cholesterol detection solution was added per well and shaken for 30–60 s at low RPM before incubation at room temperature for 1 h. Luminescence was read using a CLARIOstar microplate reader and normalized to a replicate resazurin-based cell viability assay.

### Combination experiment and synergistic calculation

In a 96-well plate, 12,000 CHP-212 and IMR-32 cells/well were plated and after overnight incubation exposed to single or combination treatment of FLIX5 with Ezetimibe, Etoposide, Vincristine, Paclitaxel, Docetaxel or Vinorelbine at indicated concentrations. The cell viability was determined by resazurin based cell viability assay. The synergistic calculation for a border range, software MacSynergy™ II [15] was used. To analyze the interaction between FLIX5 and the compounds in the indicated concentrations, the coefficient drug interaction (CDI) was calculated. The CDI was calculated as follow: CDI = AB/(A x B) where AB is equivalent to the cell viability of the combination treatment of FLIX5 and respective compound. A and B is equivalent to the cell viability of the single treatment of FLIX5 or the compound. To analyze the interaction as synergistic, additive or antagonistic. A CDI value of >1, =1 or <1 indicates the drugs are antagonistic, additive or synergistic respectively and if the CDI value is below 0.7 the drugs are significantly synergistic [16].

### Single cell sequencing and analysis

#### Single-cell RNA sequencing library preparation

Single-cell capture, lysis, reverse transcription, and pre-amplification were performed in the single-cell system following the manufacturer’s protocols by Eukaryotic Single-Cell Genomics Facility (Karolinska Institute, Sweden). Libraries were sequenced using the Illumina NovaSeq 6000 System.

#### Single-cell RNA-Seq analysis

Data preprocessing and analysis were performed using the 10x Genomics CellRanger 6.0.2 software [17]. Cell barcodes were demultiplexed, and reads were aligned to the GRCh38 human reference transcriptome using the STAR aligner. Cell identification was based on cell barcodes, with a threshold set to remove low-quality cells. Cells with fewer than 100 counts, fewer than 100 detected genes, or mitochondrial content exceeding 0.3% were filtered out. DoubletFinder [18] was applied to distinguish between empty droplets and droplets containing single cells. Following this, the raw gene count matrix from the CellRanger output was filtered to remove unqualified cells.

Downstream analysis was performed using the Seurat 4.2.1 R package [19]. Expression normalization was achieved using the LogNormalize() function in Seurat, adjusting for variations in library size and cell cycle effects. To account for unwanted sources of variation, such as the number of UMIs, mitochondrial content, and cell cycle differences, a linear regression model was applied during gene scaling and centering. Cell cycle effects were further mitigated by employing the “Alternate Workflow” in Seurat, utilizing previously published G2/M and S-phase gene signatures to remove confounding differences. Expression values were then scaled across all cells within the dataset, and scaled z-score residuals (relative expression) were used for visualization dimensionality reduction and clustering. Principal component analysis (PCA) was conducted on all expressed genes, with significant principal components serving as inputs for nonlinear dimensionality reduction techniques, such as UMAP, and cell clustering. Differential expression analysis was carried out using the FindAllMarkers function, employing the Wilcoxon rank-sum test with a false discovery rate (FDR) threshold of 0.05. Genes with a detection rate difference of 0.1 or greater between clusters were included in the differential testing. For pathway enrichment analysis, Hallmark pathways were analyzed using the fgsea R package. Genes were ranked based on their area under the curve (AUC) values, which were calculated using the presto R package. Pathways were selected for enrichment analysis based on an FDR < 0.05.

### Connectivity map (C-MAP) analysis

CHP-212 cells were treated with FLIX5 for 48 h at the 48h-IC50 concentration. Following the treatment, proteomics sample preparation was conducted using the previously described PISA method. Briefly, protein concentration of supernatants was measured by microBCA equal amounts of peptides resulting from different samples were labeled using TMTpro 16-plex technology and one final multiplex sample were obtained from the experiment. The last was fractionated off-line by microscale preparative reverse chromatography into fractions to be run on LC-MS. LC-MS of all fractions was conducted using Nano-LC-ESI_MS/MS on a high resolution Orbitrap Exploris 480 instrument. Proteome Discoverer was used for database search and quantification against the Uniprot Homo sapiens (Human) protein database UP000005640. Datasets were normalized on total intensity and normalization on average of controls, and relative p-values (student’s *t* test) for statistical significance of variation. Proteome discoverer search with quantification analysis of the spectra generated by LC-MS/MS of fractionated sample was able to identify and quantify the following:

**Table:** 

Peptide sequences ID (identified with TMT labeling)	221,905
Protein ID after contaminants removal across all replicates	11,485
Protein ID and quantified with ≥ 2 unique peptides across all replicates	9849

The complete list of identified peptides after 48 h treatment can be found in Supplementary Table 3. The expression patterns following FLIX5 treatment, including the top 100 upregulated and downregulated gene symbols, were compared to the Connectivity Map database to identify perturbations with similar gene expression responses.

### Apoptosis assay

To analyze apoptosis, an M30 Apoptosense ELISA (VLVbio, Sweden, #10011) was performed on neuroblastoma cell lines CHP-212 and IMR-32. In a 96-well plate, 25,000 cells of each respective cell line were plated per well. After overnight incubation, CHP-212 and IMR-32 cells were exposed to FLIX5 for 24 h. Following drug exposure, 10 µL RIPA lysis and extraction buffer (Thermo Fisher Scientific, Waltham, MA, USA, Cat. #89901) was added to each well and shaken for 5 min. The M5 coated microplate was prepared, and the M30 assay was performed as follow; 25 µL of M30 standard A-G and sample was added per well, followed by adding 75 µL of diluted M30 per well and incubated for 4 h on plate shaker at 600RPM. The plate was washed manually with 5*100 µL of prepared wash water. 200 µL TMB substrate was added per well and incubated for 20 min in darkness followed by addition of 50 μL stop solution Absorbance was measured at 450 nm using a CLARIOstar microplate reader. The ccK18 signal was calculated based on the standard curve and normalized to the cell viability of duplicate-plated cells using a resazurin cell viability assay. To confirm apoptosis, the Caspase-3/7 Cell Event Kit (Invitrogen, Cat. #C10723) was used to test CHP-212 cells exposed to FLIX5 and 0.5 μM staurosporine for 4 h. After exposure, cells were dissociated with trypsin and incubated with 5 μM caspase-3/7 detection reagent in 1 mL medium for 45 min. Detection of the GFP signal was performed using the Countess 3FL (Invitrogen, Cat. #AMQAF2000) equipped with the EVOS™ Light Cube, GFP 2.0 (Invitrogen, Cat# AMEP4951), in a 1:1 ratio with trypan blue (Thermo Fisher Scientific, Waltham, MA, USA, Cat. #T10282). Acquired images were analyzed using the “Find Edges” command in ImageJ.

### Tumor organoids

Neuroblastoma PDX-derived cells LU-NB-1 and LU-NB-2, obtained from Professor Daniel Bexell (ethic approve ref. no. 2016/1055, 2018/155), were cultured in serum-free medium as previously described [20, 21]. Briefly, the cells were grown as tumor organoids in a 3:1 mixture of Dulbecco’s modified Eagle’s low glucose medium (ThermoFisher Scientific, #21885108) and Ham’s F-12 Nutrient Mix, GlutaMAX™ Supplement (ThermoFisher Scientific, #31765027), supplemented with 1% penicillin/streptomycin (ThermoFisher Scientific #15140122), 2% B27 without vitamin A (ThermoFisher Scientific, #12587010), 40 ng/ml fibroblast growth factor (Peprotech, #100-18B), and 20 ng/ml epidermal growth factor (Peprotech, AF-100-15). The cells were regularly verified by single-nucleotide polymorphism (SNP) analysis and screened for mycoplasma. A Whitley H35 Hypoxystation (Don Whitley Scientific) was used to maintain a 5% CO2 environment. For culture maintenance organoids we dissociated with Accutase (Sigma Aldrich, #A6964) with further passaging at 1 ml cells/10 ml density.

For drug treatment 40 000 cells/well were seeded in opaque white plates (ThermoFisher Scientific, #136101), incubated 48 h to form organoid-like structures and treated with FLIX5, Vincristine and Vinorelbine or DMSO control (0.02%). After determining IC50 for the abovementioned compounds combinatorial treatment of FLIX5+Vincristine and FLIX5+Vinorelbine in a form of treatment matrix were performed in both organoid models. To determine cell viability cellular ATP was assessed with CellTiter-Glo 2.0 assay (Promega, #G9243) according to the manufacturer. Luminescence was measured at integration time of 1 second using CLARIOstar® plate reader (BMG Labtech). To detect and calculate the synergism of the combinatorial treatment, we used the web-based program SynergyFinder [15] and the software MacSynergy™ II [22]. Using SynergyFinder, there is no particular threshold to define a good synergy score, since combination synergy is highly context-specific. synergy score: Less than -10: the interaction between two drugs is likely to be antagonistic; from -10 to 10: the interaction between two drugs is likely to be additive; and larger than 10: the interaction between two drugs is likely to be synergistic. Using MacSynergy™ II, synergy score>0, synergy score: Less than 0: the interaction between two drugs is likely to be antagonistic; 0: the interaction between two drugs is likely to be additive; and larger than 0: the interaction between two drugs is likely to be synergistic.

### Statistical analysis

Statistical parameters and tests are reported in the Figures and corresponding Figure Legends. Statistical analysis was done using GraphPad Prism (GraphPad Software Inc). Using nonlinear regression, [Inhibitor] vs. normalized response analysis, the half-maximal inhibitory concentration (IC50) was calculated. Results were considered statistically significant when the *p* value was less than 0.05 (*p* < 0.05).

## Results

### A chemical library screening uncovers FLIX5, a small molecule toxic to neuroblastoma and medulloblastoma cells regardless of MYCN status

**Fig. 1:**
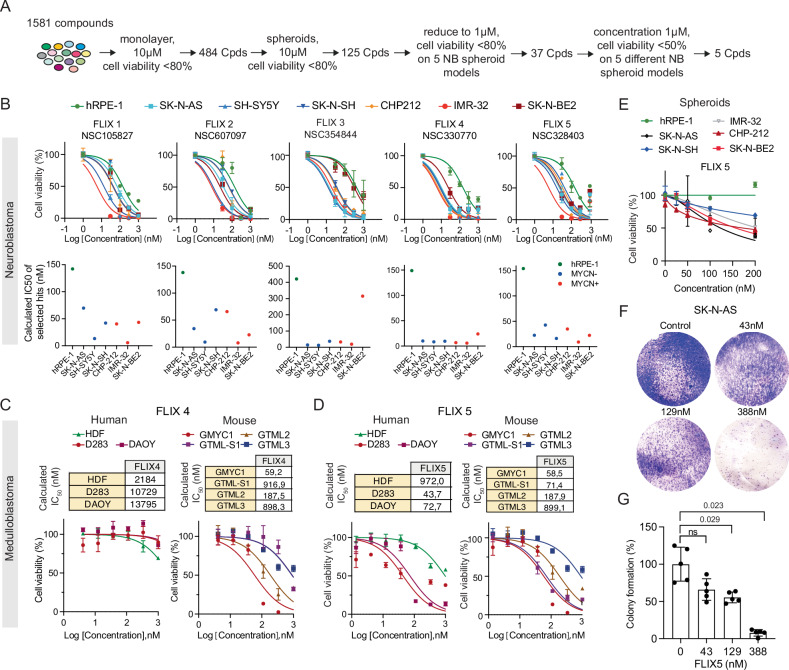
Screening of chemical libraries identifies toxic agents for neuroblastoma and medulloblastoma cells, regardless of MYCN status, in both 2D and 3D conditions. **A** Workflow of the cell-based drug screening. A total of 1581 compounds were assessed in a cell-based screening in 96-well plates in both 2D- and 3D-conditions to find compounds that exhibited pan toxicity to neuroblastoma cells. Five compounds, FLIX1 (NSC105827), FLIX2 (NSC607097), FLIX3 (NSC354844), FLIX4 (NSC330770), and FLIX5 (NSC328403), were identified showing >50% cytotoxicity to 5 different neuroblastoma (NB) spheroids with or without MYCN overexpression at 1 µM after 72 h treatment. **B** Dose-response of the 5 identified compounds, FLIX1– FLIX5, which show a greater toxicity on 6 neuroblastoma cell lines compared to immortalized hTERT RPE-1 cells in 2D-condition. One representative experiment with 3 technical replicates is shown (mean ± SD). **C** Dose-response of FLIX4 on human medulloblastoma cells and mouse derived medulloblastoma cells. One representative experiment with 3 technical replicates is shown (mean ± SD). **D** Dose-response of FLIX5 on human medulloblastoma cells and mouse derived medulloblastoma cells. One representative experiment with 3 technical replicates is shown (mean ± SD). HDF: human dermal fibroblast (normal control cell line). DAOY human medulloblastoma cells, D283 human medulloblastoma cells, high express MYC. GMYC1: mouse medulloblastoma cells, high express MYC [10]. GTML-S1: mouse medulloblastoma cells, high express MYCN [11]; GTML2: mouse medulloblastoma cells, high express MYCN [46]; GTML3: mouse medulloblastoma cells, high express MYCN [46]. **E** Dose-response of FLIX5 on neuroblastoma and immortalized hTERT RPE-1 spheroids. One representative experiment with 3 technical replicates is shown (mean ± SD). **F** Colony formation assay of SK-N-AS spheroids treated with FLIX5 at concentrations of 43 nM, 129 nM, and 388 nM for 72 h. After exposure, the spheroids were dissociated, and the cells were cultured until cells reached confluency under control conditions. **G** Quantification of the colony formation assay in (**F**) revealed statistically significant differences between the control and FLIX5 treatments at concentrations of 129 nM and 388 nM. One representative experiment with 5 technical replicates is shown (mean ± SD; *t* test, *p* < 0.05 is considered significant).

To discover undisclosed drug targets for neuroblastoma, we initiated a chemical screening by first subjecting 1581 compounds, selected from a vast pool of around 140,000 chemicals (NCI Diversity Set VII), on monolayer cultures to eliminate non-toxic compounds to neuroblastoma cells. Subsequently, the remaining compounds were tested on various spheroid models at different concentrations (10 and 1 µM). Following multiple rounds of selection, we identified five compounds, FLIX1 (NSC105827), FLIX2 (NSC607097), FLIX3 (NSC354844), FLIX4 (NSC330770), and FLIX5 (NSC328403). The screening process is detailed in Fig. 1A and Supplementary Fig. 1A, B. To ensure that the observed toxicity is cancer cell-specific, we then compared the cell viability between hTERT RPE-1 (hTERT-immortalized retinal pigment epithelial) cells and 6 neuroblastoma cells with or without MYCN overexpression. The compounds FLIX1 and FLIX2 displayed strong toxicity towards neuroblastoma cells. SK-N-BE2 cells, a cell line from a patient after repeated courses of chemotherapy and radiotherapy, and more resistant to additional chemo-treatment [23], showed a greater resistance to FLIX3. Compounds FLIX4 and FLIX5 exhibited a greater toxicity against neuroblastoma cells compared to compound FLIX1 and FLIX2, while maintaining a substantial therapeutic window between hTERT RPE-1 (hRPE1) and neuroblastoma cells (Fig. 1B). Based on the drug sensitivity data we opted to focus on FLIX4 and FLIX5.

The primary focus of our study is neuroblastoma, moreover, we also aim to explore the broader potential of the compound for treating other pediatric tumors. To this end, we tested FLIX4 and FLIX5 on two human and four mouse-derived medulloblastoma cell lines. These cells are known for their high resistance to chemotherapy. While FLIX4 exhibited promising efficacy against 6 neuroblastoma cell lines, its potency faltered against human medulloblastoma cells (Fig. 1C). FLIX5 still displayed desirable cytotoxicity against both human and mouse-derived medulloblastoma cells, alongside a significant therapeutic window to HDF (human dermal fibroblast) cells (Fig. 1D). Considering all the findings, we concluded to proceed with FLIX5 as the candidate. Further testing of FLIX5 on various neuroblastoma spheroid models revealed sustained strong toxicity toward neuroblastoma spheroids at nanomolar concentrations, while showing no toxicity on hRPE1spheroids, even up to 5000 nM (Fig. 1E, Supplementary Fig. 1C,D). Additionally, colony formation assays indicated that after the removal of FLIX5 from spheroids, dispensed cells significantly lose the ability to regrow above 129 nM (Fig. 1F, G, Supplementary Fig. 1E, F).

### EPLIN protein has been identified as a target of FLIX5

**Fig. 2:**
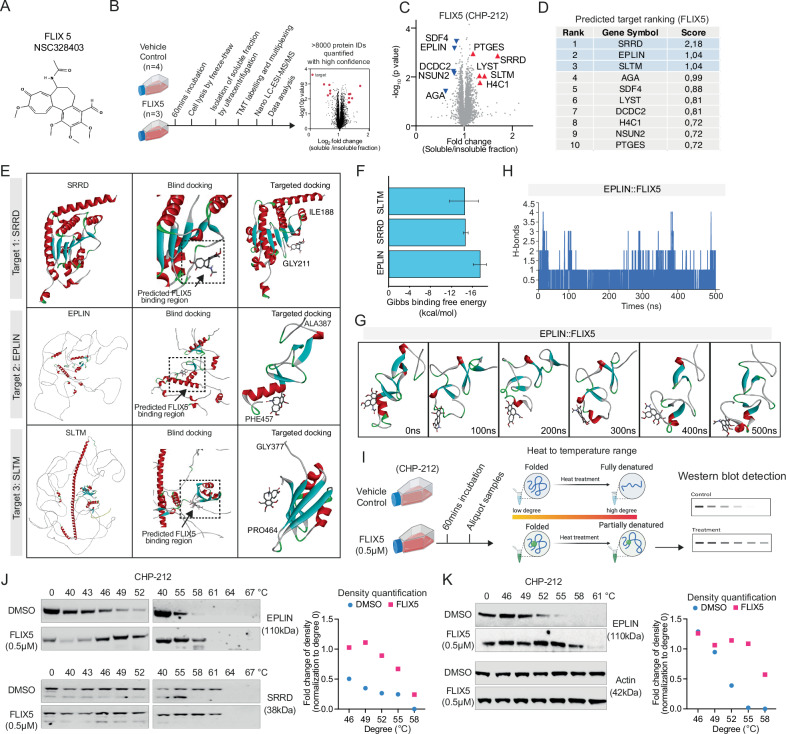
FLIX5 targets EPLIN in neuroblastoma cells. **A** The chemical structure of FLIX5 (NSC328403). **B** An illustration of workflow of the proteome integral solubility alteration (PISA) assay. The figure was created with Biorender.com. **C** The volcano plot illustrates the PISA results. The red triangle marks significantly upregulated proteins in FLIX5-treated samples while the blue triangle marks significantly downregulated proteins, based on fold change from soluble fraction to insoluble fraction. DMSO (*n* = 4) and FLIX5 (*n* = 3). The calculated statistical significance (*p* < 0.05, *t* test). **D** The predicted target ranking displays the top 10 altered proteins identified in (**C**). Score value was calculated using the formula: -Log10 (*p* value) $\times$ Log2 (fold change) in the PISA result. The calculated statistical significance (*p* < 0.05, *t* test). **E** Computational molecular-docking analysis of the binding possibility of FLIX5 to SRRD, EPLIN, and SLTM. The AlphaFold algorithm was utilized for protein structure prediction, followed by an unbiased docking approach to predict the binding regions of the target ligand. Subsequent re-docking with the target ligand validated the initial predictions of FLIX5 binding to the top 3 hit proteins: SRRD, EPLIN, and SLTM, as shown in (**D**). **F** Solvent-based Gibbs binding free energy calculated after a short (1 ns) molecular dynamic simulation between FLIX5 and SLTM, SRRD or EPLIN. **G** Snapshots taken at indicated time points to predict the binding stability of FLIX5 at the predicted site in EPLIN. To assess the binding stability of the FLIX5 complex with EPLIN a protein-ligand complex was subjected to a 500 ns (0.5 µs) molecular dynamics simulation using the GROMACS simulation package. Atomistic MD simulation demonstrated that the target ligand binds stably and comfortably to the EPLIN protein throughout the 500 ns simulation. **H** The number of h-bonds between EPLIN and FLIX5 throughout the 500 ns simulation. **I** An illustration of the workflow of the cellular thermal shift assays (CETSA). The figure was created with Biorender.com. **J** Left: CETSA were employed to detect the stability of EPLIN and SRRD in samples treated with either DMSO or FLIX5 (0.5 µM) for one hour using CHP-212 cells. The temperature tested ranged from 40 to 67 °C, with intervals of 3 °C. Right: quantification of band densities in the Western blots at the indicated temperatures. **K** Left: the CETSA experiment was further validated in samples treated with either DMSO or FLIX5 (0.5 µM) for one hour using CHP-212 cells. The temperature tested ranged from 46 to 61 °C, with intervals of 3 °C. Right: quantification of band densities in the Western blots at the indicated temperatures. The CETSA experiment was repeated at least three times independently.

To understand why FLIX5 (Fig. 2A) exhibits potent toxicity, we conducted a proteome integral solubility alteration (PISA) experiment. PISA provides deep and quantitative proteomic analysis of changes in the amount of the soluble and insoluble protein fractions of detected proteins. In our experiment, it is important to ensure that the cells express the target of the compound. Since FLIX5 is potent against all neuroblastoma cells, we assume that all of these cells express the target proteins. In addition, since downstream thermal shift assays require a large number of cells with strong adhesion in our experiment, we chose CHP-212 for this purpose [13, 24]. CHP-212 cells were exposed to FLIX5 for 60 min, followed by the collection of cell pellets for liquid chromatography-mass spectrometry (LC-MS) analysis (Fig. 2B). Analysis of the soluble and insoluble protein fractions under FLIX5 and DMSO treatment conditions revealed significant alterations in protein soluble/insoluble fraction (Fig. 2C, Supplementary Fig. 2A and Supplementary Table 1), with the top 10 predicted targets listed in Fig. 2D.

To confirm the potential target of FLIX5 from the top 10 target candidates (Fig. 2D), we utilized the AlphaFold algorithm for protein structure prediction, followed by an unbiased docking approach to predict the binding regions of the target ligand. Subsequent re-docking with the target ligand validated the initial predictions (Fig. 2E). We further evaluated the binding stability through solvent-based Gibbs binding free energy calculations (Fig. 2F), suggesting that FLIX5 prefers to bind to EPLIN compared to SRRD and SLTM, even though SRRD ranked first in the predicted target ranking list (Fig. 2D). To assess the binding stability of FLIX5 with EPLIN, a protein-ligand complex was subjected to a 500 ns (0.5µs) molecular dynamics simulation using the GROMACS simulation package. Atomistic MD simulation demonstrated that the target ligand binds stably and comfortably to the EPLIN protein throughout the 500 ns simulation. Snapshots taken at different time points consistently showed that the ligand binds stably at the predicted site in the EPLIN protein (Fig. 2G). Simulation trajectory analysis indicated that ligand RMSD (root mean square deviation) remained stable throughout the 500 ns simulation, suggesting minimal fluctuation of the ligand during the simulation. Similarly, the number of hydrogen bonds between FLIX5 and EPLIN was stable throughout the simulation, indicative of stable ligand binding (Fig. 2H). Furthermore, the Gibbs binding free energy assessment for trajectories covering the entire 500 ns simulation depicted a favorable binding free energy for FLIX5 toward the EPLIN protein is VDWAALS (Van der Waals forces) and GGAS (Guanine-Guanine Adenine-Adenine Stacking) (Supplementary Fig. 2B). Collectively, these results predict that FLIX5 exhibits a preference for EPLIN over SLTM and SRRD and binds stably to EPLIN.

The cellular thermal shift assay (CETSA) is a technique used to investigate the interaction between proteins and small molecules or other ligands in live cells. It operates on the principle that the thermal stability of a protein can change upon binding to a ligand, making it particularly useful for validating drug targets within their physiological context [14]. To further validate our computational predictions, we performed CETSA on samples exposed to either DMSO or FLIX5 for 60 min (Fig. 2I). Our result showed that EPLIN exhibited a greater stability in cells treated with FLIX5 compared to SRRD (Fig. 2J), indicating that EPLIN is a preferred target of FLIX5. We further validated the finding that FLIX5 targets EPLIN through additional experiments (Fig. 2K and Supplementary Fig. 2C), accompanied by corresponding density quantification. Collectively, our results suggest that EPLIN exhibits greater stability in cells treated with FLIX5 at temperatures ranging from 52–55 °C. Altogether, we conclude that protein EPLIN is a target of FLIX5.

### Reduction in EPLIN expression promotes cancer cell growth and is associated with poorer overall survival

**Fig. 3:**
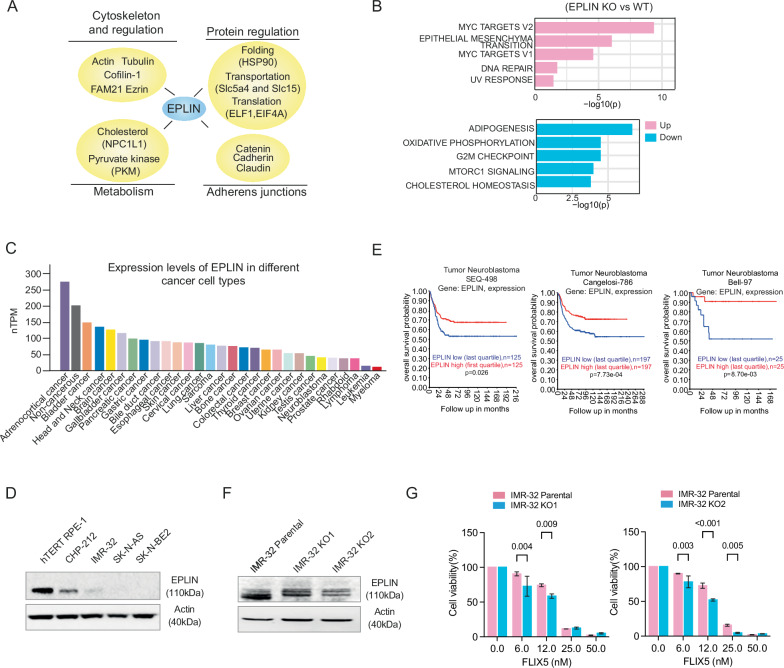
Reduction in EPLIN expression promotes cancer cell growth and is associated with poorer overall survival. **A** Illustration displaying the proteins in direct interaction with EPLIN in various key cellular biological functions. The data resource originally came from studies [25, 29]. **B** The top five significantly Up- and downregulated hallmark pathways in the EPLIN KO groups. The full list of altered hallmark pathways with significance can be found in Supplementary Table 4. **C** The expression levels of EPLIN in various cancer cell types and noncancer cells were analyzed using publicly available data from the Protein Atlas Cohort. **D** Immunoblots detecting EPLIN in immortalized hTERT RPE-1 and various neuroblastoma cells. Actin is loading control. **E** The overall survival rates of neuroblastoma patients with high and low EPLIN gene expression (the first quartile vs. the last quartile) were examined using Kaplan-Meier survival curves in SEQC498 (*p* = 0.0260), Cangelosi-786 (*p* = 0.0007), and Bell-97 datasets (*p* = 0.0087), which include 498, 786, and 97 neuroblastoma samples, respectively. Statistical analyses were conducted using the log-rank test, with data sourced from publicly available patient cohorts on the R2 microarray analysis and visualization platform. **F** The expression levels of EPLIN in IMR-32 parental and KO clones. Actin is loading control. **G** Response of IMR-32 parental, KO1, and KO2 clones to the indicated concentrations of FLIX5 for 72 h. One representative experiment with 3 technical replicates is shown (mean ± SD, *t* test, *p* < 0.05 is considered significant).

EPLIN (Epithelial Protein Lost In Neoplasm), also known as LIMA1, is a key regulator of cytoskeletal dynamics, organization, cell motility, growth and metabolism (Fig. 3A). We analyzed the public dataset (E-MTAB-10301) containing RNA-Seq data from conditions of EPLIN overexpression and knockout in both mouse and human models [25], and selected genes that showed consistent results across both OE and KO groups (upregulated in OE and downregulated in KO, and vice versa). The over-representation tests for pathway enrichment indicated that lacking EPLIN expression exhibits an upregulation of MYC-targeted pathways and epithelial-mesenchymal transition, while showing downregulation of oxidative phosphorylation and cholesterol homeostasis (Fig. 3B, Supplementary Table 4). EPLIN is predominantly expressed in human epithelial cells and is frequently downregulated in cancerous cells ([26] and Fig. 3C). Indeed, we noticed that neuroblastoma cells express much lower levels of EPLIN compared to hTERT RPE-1 cells (Fig. 3D). Furthermore, in three out of four independent neuroblastoma datasets (Asgharzadeh-249 dataset, *p* = 0.293; SEQC-498 dataset, *p* = 0.026; Cangelosi-786 dataset, *p* < 0.001 and Bell-97 dataset, *p* = 0.008), lower EPLIN expression was significantly associated with poorer overall survival (Fig. 3E and Supplementary Fig. 3A). To better understand the role of EPLIN in neuroblastoma cells, we generated IMR-32 cell clones with reduced EPLIN expression (Fig. 3F) and treated them with FLIX5 at the indicated concentrations (Fig. 3G). These low-EPLIN-expressing cells exhibited increased sensitivity to FLIX5, suggesting that FLIX5 causes EPLIN-dependent cytotoxicity.

In summary, we propose that in neuroblastoma, reduced expression of EPLIN acts as a double-edged sword. On one hand, low levels of EPLIN expression can promote cancer initiation and progression. On the other hand, cancer cells with low EPLIN expression may become more dependent on the remaining EPLIN to maintain the cellular activities regulated by EPLIN. Further disruption of EPLIN function, or its interactions with proteins such as actin, tubulin, or NPC1L1, may lead to increased toxicity in cancer cells relative to normal cells.

### FLIX5 induces mitochondrial dysfunction and heightens dependency on lipid metabolism

**Fig. 4:**
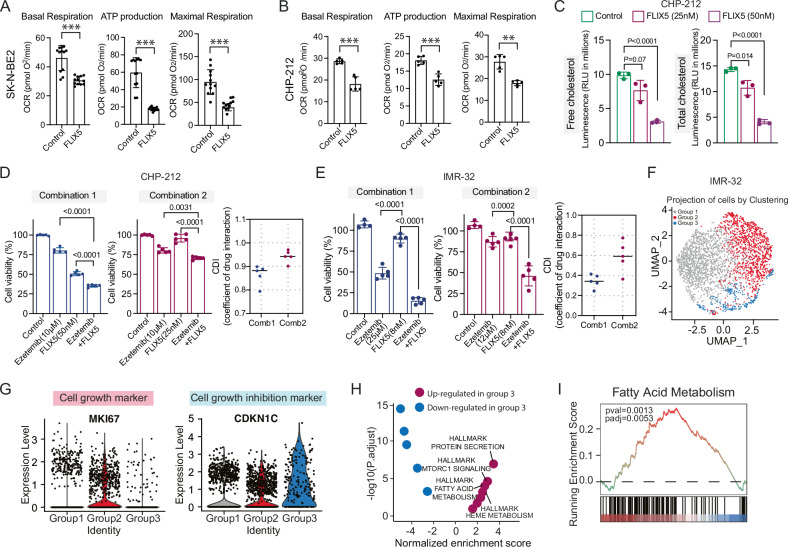
FLIX5 impairs mitochondrial function and increases the dependency of neuroblastoma cells on lipid metabolism. **A** Comparison of basal respiration, ATP production, and maximal respiration levels in SK-N-BE2 cells treated with DMSO or 50 nM FLIX5 overnight using an automatic flux analyzer. For SK-N-BE2, one representative experiment with 12 technical replicates is shown. For CHP-212 cells, one representative experiment with 5 technical replicates is shown (mean ± SD, ** *p* < 0.01, *** *p* < 0.001; unpaired two-tailed *t* test). **B** Comparison of basal respiration, ATP production, and maximal respiration levels in CHP-212 cells treated with DMSO or 50 nM FLIX5 overnight using an automatic flux analyzer. For SK-N-BE2, one representative experiment with 12 technical replicates is shown. For CHP-212 cells, one representative experiment with 5 technical replicates is shown (mean ± SD, ** *p* < 0.01, *** *p* < 0.001; unpaired two-tailed *t* test). **C** Intracellular levels of free and total cholesterol in cells treated with DMSO or FLIX5. One representative experiment with 3 technical replicates is shown (mean ± SD, unpaired two-tailed *t* test, *p* < 0.05 is considered significant). **D** Evaluation of the combination effect of FLIX5 with Ezetimibe in CHP-212 cells for 72 h. In combination 1, the FLIX5 concentration was 50 nM, and in combination 2, the FLIX5 concentration was 25 nM. The concentration of Ezetimibe remained the same at 10 µM in both combinations. One representative experiment with 5 technical replicates is shown (mean ± SD, unpaired two-tailed *t* test, *p* < 0.05 is considered significant). **E** Evaluation of the combination effect of FLIX5 with Ezetimibe in IMR-32 cells for 72 h. In combination 1, the Ezetimibe concentration was 25 µM, and in combination 2, the Ezetimibe concentration was 12 µM while FLIX5 concentration was the same at 8 nM for both combinations. One representative experiment with 5 technical replicates is shown (mean ± SD, unpaired two-tailed *t* test, *p* < 0.05 is considered significant). CDI: the coefficient drug interaction. A CDI value of >1, =1 or <1 indicates the drugs are antagonistic, additive, or synergistic, respectively and if the CDI value is below 0.7 the drugs are significantly synergistic [16]. **F** UMAP visualization of scRNA-sequencing data of IMR-32 spheroid-model identifying the presence of three cell clusters, Group 1 in gray, Group 2 in red and Group 3 in blue. **G** Bar plot of gene expression for the cell-cycle active marker MKI67 and cell growth arrest marker CDKN1C in the three cluster groups identified in (**G**). **H** Scatterplot depicting significantly altered hallmark pathways in Group 3. Blue dots represent the top significantly downregulated pathways, and purple dots represent the top significantly upregulated pathways. **I** GSEA enrichment profile of the significantly altered hallmark fatty acid metabolism pathway (pval = 0.0013, padj = 0.0053) identified in Group 3.

In previous studies and our analysis, EPLIN knockout cells exhibited impaired mitochondrial activity ([25] and Fig. 3B). Given that FLIX5 targets the EPLIN protein, we asked whether FLIX5 could as well impact mitochondria. To address this, we conducted a mitochondrial stress test to measure the oxygen consumption rate in SK-N-BE2 and CHP-212 cells treated with DMSO or FLIX5. We observed a significant reduction in basal, ATP-linked, and maximal respiration in FLIX5-treated cells (Fig. 4A, B). Mitochondria serve as the cellular energy powerhouse, and their impairment could lead to an energy crisis, as previously observed [27, 28]. Interestingly, intracellular ATP levels did not decrease following FLIX5 treatment (24 or 48 h, Supplementary Fig. 4A) nor under EPLIN knockout conditions (Supplementary Fig. 4B). This suggests the presence of potential energy compensation mechanisms in response to FLIX5-induced mitochondrial disturbances. EPLIN was reported to directly interact with NPC1L1 (Fig. 3A and Supplementary Fig. 4C), a key player in cholesterol transportation targeted by ezetimibe [29]. Cholesterol can be synthetized de novo from acetyl coenzyme A (acetyl-CoA) via the activity of >20 enzymes catalyzing complex reactions involved in lipid metabolism [30]. We conducted measurements of both free and total cholesterol in cells treated with and without FLIX5. Our analysis revealed a decrease in both free and total cholesterol levels in cells exposed to FLIX5 for 48 h (Fig. 4C). Additionally, we observed that neuroblastoma cells exhibited synergistic sensitivity to FLIX5 when combined with ezetimibe, which targets NPC1L1 and blocks intestinal cholesterol absorption (Fig. 4D, E). This suggests an increased dependency on cholesterol and lipid metabolism in FLIX5-treated cells. Together, we conclude that the cholesterol biosynthesis pathway acts as a compensatory mechanism for mitochondrial deficiency caused by FLIX5.

Moreover, we identified a distinct cell population (Group 3) using unsupervised two-dimensional uniform manifold approximation and projection (UMAP) in single-cell RNA-Seq analysis of IMR-32 spheroids (Fig. 4F, Supplementary Fig. 4D). Group 3 was characterized by growth arrest, with lower expression of the cell growth marker Ki67 and higher expression of the cell growth inhibition marker CDKN1C (Fig. 4G). The presence of growth-arrested cancer cells presents a significant therapeutic challenge, as these cells often resist to traditional cell cycle-targeted therapies. Interestingly, this growth-arrested Group 3 exhibited upregulation in fatty acid metabolism (Fig. 4H, I, Supplementary Fig. 4E). These findings may help explain why FLIX5 exhibits significant toxicity in spheroids containing both proliferating and cell-cycle–arrested populations, potentially due in part to its targeting of cholesterol regulation pathways.

### FLIX5 induces apoptosis

**Fig. 5:**
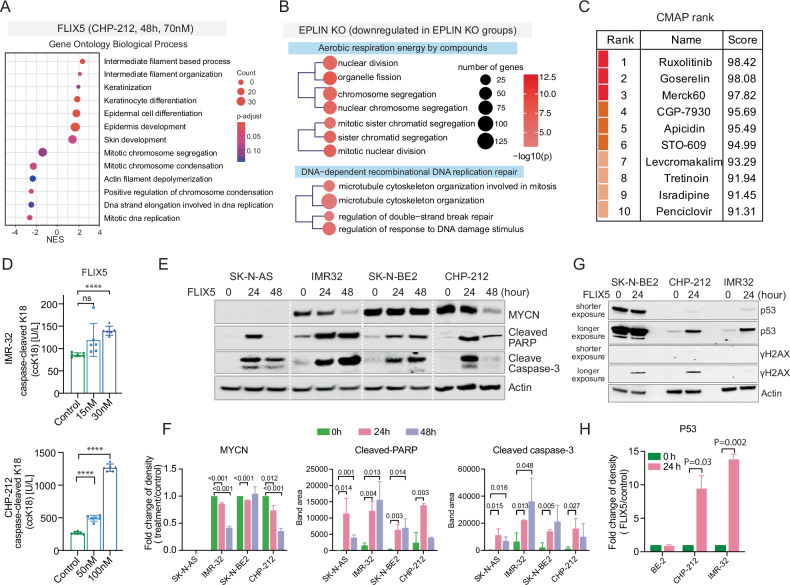
FLIX5 induces apoptosis as the primary mechanism of cell death. **A** Changes in gene ontology biological processes in CHP-212 cells treated with FLIX5 (70 nM, 48 h, IC50) for 48 h. The full list of altered hallmark pathways with significance can be found in Supplementary Table 5. **B** Downregulated gene ontology biological processes with significance in EPLIN KO groups. The full list of altered hallmark pathways with significance can be found in Supplementary Table 6. **C** CMAP analysis displaying the top 10 most relevant compounds, to FLIX5 with known cell death mechanisms. A CMAP-score of 100 indicates identical mechanism. **D** Levels of caspase-cleaved K18 (ccK18) in IMR-32 and CHP-212 cells treated with DMSO or FLIX5 with indicated concentrations for 48 h. One representative experiment with 6 technical replicates is shown (mean ± SD, unpaired two-tailed *t* test, ns, *p* > 0.05; ****, *p* < 0.0001). **E** Immunoblots detecting markers of apoptosis in neuroblastoma cells treated with DMSO or FLIX5 for 24 and 48 h. Cleaved caspase-3 and cleaved PARP are markers of apoptosis. MYCN status is shown, with actin used as the loading control. **F** Measurement of band density of immunoblots, in (**E**), for indicated proteins. The data was obtained from immunoblots repeated across three independent experiments (mean ± SD, unpaired two-tailed *t* test, *p* < 0.05 is considered significant). **G** Immunoblots were conducted to detect changes in the expression of p53 and γH2AX in cells treated with either DMSO or FLIX5 for 24 h. Actin is loading control. **H** Measurement of band density of immunoblots, in (**G**), for p53. Immunoblots repeated across three independent experiments. Data are presented as mean ± SD, unpaired two-tailed *t* test, *p* < 0.05 is considered significant.

To delve into the mechanism of action underlying FLIX5, we first employed expression proteomics using Nano-LC-ESI-MS/MS on CHP-212 cells treated with FLIX5 for 48 h. From this analysis, we identified 50 proteins significantly upregulated (fold change FLIX5/DMSO > 1.5, *p* value ≤ 0.05, Supplementary Fig. 5A, C), and 41 proteins significantly downregulated (fold change FLIX5/DMSO < 0.85, *p* value ≤ 0.05, Supplementary Fig. 5B, D) in FLIX5-treated cells. Notably, methionine aminopeptidase 2 (METAP2), primarily responsible for regulatory functions related to translational initiation and cellular growth [31], has been a tentative drug target for the treatment of cancer [32] was prominently reduced in FLIX5-treated samples (fold change FLIX5/DMSO = 0.49, *p* value = 0.03, Supplementary Fig. 5B). Gene Ontology analysis revealed upregulation of processes related to intermediate filament organization and downregulation of pathways involving mitotic chromosome segregation, condensation and DNA replication, processes crucial for cell division [33]. Given that EPLIN, also known as LIMA1, is a critical component in regulating cytoskeletal dynamics (actin and β-catenin) and influencing alterations in cell motility and cell-cell adhesions [26, 34], the downregulation of actin filament depolymerization may be linked to the fact that FLIX5 targets EPLIN (Fig. 5A, Supplementary Table 5). We further investigated the alterations in Gene Ontology biological processes in EPLIN KO groups. We found that microtubule cytoskeleton organization, regulation of response to DNA damage stimulus, and pathways involved in chromosome segregation regulation were also downregulated in EPLIN KO groups compared to the WT group (Fig. 5B, Supplementary Table 6). This suggests that the response to FLIX5 induces similar biological process changes as those observed in the absence of EPLIN.

The common types of cell death include apoptosis (type I), autophagy (type II), and necrosis (type III) [35]. Since we did not observe autophagy following FLIX5 treatment (Supplementary Fig. 5E), we hypothesized that FLIX5 might induce cell death through apoptosis or necrosis. To delve deeper into the mechanism of cell death induced by FLIX5, we conducted a connectivity map (CMAP) analysis, which compares the expression profiles of approximately 5000 small-molecule compounds [36]. Given that our data were generated from a 48-h treatment of FLIX5, a time point at which cell death had already been initiated, we hypothesized that signatures with high similarity in the CMAP analysis could provide insights into the mechanism of cell death rather than the target indication. Indeed, the top three drugs identified in the CMAP analysis—JAK Inhibitor ruxolitinib [37], pituitary gonadotropin secretion inhibitor goserelin [38], and HDAC1/2 inhibitor Merck60 [39]—have distinct cellular targets, yet all induce apoptosis (Fig. 5C). Building on our prior findings, which revealed no induction of autophagy in FLIX5-treated cells, we inferred that apoptosis serves as the principal mechanism of cell death triggered by FLIX5. To confirm our hypothesis, we performed the apoptosis M30 assay, detecting the marker of apoptosis caspase-cleaved k18. We observed an increase in caspase-cleaved k18 levels in both IMR-32 and CHP-212 cells after 48 h of FLIX5 treatment (Fig. 5D). An apoptosis assay targeting caspase 3/7 showed a higher proportion of caspase 3/7 stained cells in FLIX5-treated samples compared to controls (Supplementary Fig. 5F). Additionally, immunoblot analysis further revealed clear induction of cleaved PARP and cleaved caspase-3, which are present when cells undergo apoptosis, in FLIX5-treated neuroblastoma cells, irrespective of MYCN status (Fig. 5E, F). In our previous studies, we identified a homeostasis mechanism in which mitochondrial function regulates MYC expression [40]. Since FLIX5 disrupts mitochondrial activity, we also hypothesized that FLIX5-induced mitochondrial impairment would lead to downregulation of MYCN expression. However, while MYCN downregulation was observed in IMR-32 and CHP-212 cells following FLIX5 treatment, it was not obviously observed in SK-N-BE2 cells (Fig. 5E, F).

EPLIN has been identified as a direct target of the p53 family, with reported interaction and cooperation with p53 [41]. We observed an upregulation of p53 in both CHP-212 and IMR-32 cells, which are *TP53* wildtype, but not in SK-N-BE2 cells harboring a *TP53* mutation (404 G > T, Cys135Phe) (Fig. 5G) [42]. This suggests a p53-independent induction of apoptosis by FLIX5. Our analysis shows that the absence of EPLIN can trigger a response to DNA damage stimuli (Fig. 5B). To further investigate, we examined the expression of γH2AX, a marker of DNA double-strand breaks (DSBs), in cells treated with FLIX5. We observed a slight increase in γH2AX levels in SK-N-BE2 and CHP-212 cells following FLIX5 treatment. However, no such induction was detected in IMR-32 cells (Fig. 5G), suggesting that FLIX5 may induce DNA double-strand breaks in a cell type–dependent manner.

### FLIX5 synergizes with conventional chemotherapy in the treatment of neuroblastoma

**Fig. 6:**
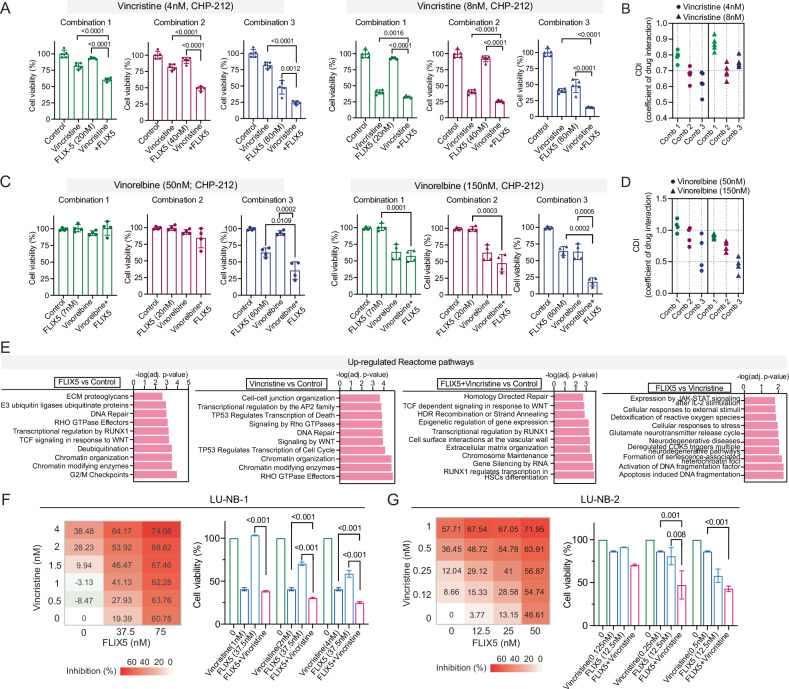
FLIX5 synergizes with vincristine or vinorelbine in the treatment of neuroblastoma. **A** The combination effect of FLIX5 at indicated concentrations with vincristine at 4 nM and 8 nM was assessed in neuroblastoma CHP-212 cells. One representative experiment with 5 technical replicates is presented (mean ± SD, unpaired two-tailed *t* test, *p* < 0.05 is considered significant). **B** Calculation of the CDI from the combination treatment assessed in (**A**). A CDI value > 1, =1 or <1 indicates the drugs are antagonistic, additive or synergistic, respectively and if the CDI value is below 0.7 the drugs are significantly synergistic. **C** Assessment of the combination effect of FLIX5 at indicated concentration with vinorelbine at 50 nM and 150 nM in neuroblastoma CHP-212 cells. One representative experiment with 5 technical replicates is presented (mean ± SD, unpaired two-tailed *t* test, *p* < 0.05 is considered significant). **D** Calculation of the CDI from the combination treatment evaluated in (**C**). A CDI value of >1, =1 or <1 indicates the drugs are antagonistic, additive, or synergistic, respectively and if the CDI value is below 0.7 the drugs are significantly synergistic. **E** The top ten significantly Up-regulated Reactome pathways in the indicated treatment groups. The list of altered pathways can be found in Supplementary Table 7. **F** Left: Drug combination matrix illustrating the interaction effects (inhibition rates) between FLIX5 and vincristine in the LU-NB-1 PDX-derived organoid model. Right: Bar chart displaying the optimal drug combination. Experiments were performed in triplicate, and results are presented as mean ± SD. Statistical analysis was conducted using an unpaired two-tailed *t* test, with *p* < 0.05 considered statistically significant. **G** Left: Drug combination matrix illustrating the interaction effects (inhibition rates) between FLIX5 and vincristine in the LU-NB-2 PDX-derived organoid model. Right: Bar chart displaying the optimal drug combination. Experiments were performed in triplicate, and results are presented as mean ± SD. Statistical analysis was conducted using an unpaired two-tailed *t* test, with *p* < 0.05 considered statistically significant.

For patients with high-risk neuroblastoma, the standard treatment regimen typically consists of dose-intensive cycles of cisplatin and etoposide, alternating with vincristine, cyclophosphamide, and doxorubicin [43]. We co-treated FLIX5 with cisplatin, etoposide, doxorubicin, vincristine, or vinorelbine and evaluated the synergistic effects using two different methods for synergy scoring. A consistent synergistic effect was observed when FLIX5 was combined with vincristine or vinorelbine. To assess synergy at specific drug concentrations, we used the Coefficient of Drug Interaction (CDI), where a CDI value below 0.7 indicates significant synergistic effect [16]. The combination with the strongest synergetic effect under vincristine treatment is 4 nM vincristine combined with 80 nM FLIX5, and the strongest synergetic effect under vinorelbine treatment is vinorelbine 150 nM combined with 60 nM FLIX5 (Fig. 6A–D, Supplementary Fig. 6A, B). We were not able to see a stable synergetic or agonistic effect when FLIX5 was combined with cisplatin, etoposide or doxorubicin. Interestingly, we observed an antagonistic effect when FLIX5 combined with docetaxel and paclitaxel (Supplementary Fig. 6A, B).

To gain deeper insights into the underlying mechanisms contributing to the synergistic effects observed in the FLIX5 and Vincristine co-treatment group, we conducted a combination experiment where synergy was noted (4 nM Vincristine with 80 nM FLIX5). Following this, we collected samples for RNA-SEQ analysis. In the GO Molecular Function analysis, it is noteworthy that the “protein heterodimerization activity” pathway was significantly upregulated in both FLIX5- and Vincristine-treated samples. Additionally, pathways related to “endopeptidase regulation” (highlighted in dark pink) were enriched only in the combination treatment, suggesting that the genes involved in these endopeptidase regulation pathways may contribute to the synergistic effect of the Vincristine and FLIX5 combination (Supplementary Fig. 6C, D, Supplementary Table 7). Strong enrichment and upregulation in various Reactome pathways were revealed (Fig. 6E, Supplementary Table 7), while enrichment of downregulated pathways was relatively weak (Supplementary Fig. 6E). Notably, “cell surface interactions at the vascular wall” and “gene silencing by RNA” were significantly enriched in the FLIX5 and Vincristine co-treatment group compared to the DMSO-treated samples. Interestingly, when comparing the effects of FLIX5 to Vincristine on cells, we found that pathways related to responses to stress and external stimuli were enriched in the FLIX5-treated samples, compared to those treated with Vincristine. Furthermore, FLIX5 induced stronger apoptosis than Vincristine (Fig. 6E), reinforcing our earlier observation that FLIX5 promotes apoptosis.

To examine the effects in patient-derived model systems, we conducted additional tests combining FLIX5 with vincristine or vinorelbine using neuroblastoma patient-derived xenograft (PDX)-derived organoids, LU-NB-1 and LU-NB-2 [20]. These models retain the chromosomal aberrations, protein markers characteristic of neuroblastoma, and tumorigenic and metastatic capabilities when reimplanted in vivo, making them especially suitable for evaluating drug efficacy [44]. Used the web-based program SynergyFinder [15] and the software MacSynergy™ II [22], we observed a stable synergistic effect of FLIX5 with vincristine (Fig. 6F, G, Supplementary Fig. 6F, G) and an additive effect with vinorelbine (Supplementary 6H, I).

### Role of EPLIN in medulloblastoma

Since FLIX5 demonstrated promising cytotoxicity against both human and mouse-derived medulloblastoma cells, while maintaining a significant therapeutic window relative to human dermal fibroblast cells (Fig. 1D), we hypothesized that EPLIN may play a similar role in medulloblastoma as previously observed in neuroblastoma. To investigate this, we generated EPLIN knockout DAOY cells using CRISPR-Cas9 (Supplementary Fig. 7A), but EPLIN was still expressed at the mRNA level. We observed a modest increase in the growth rate of EPLIN KO clones compared to the parental cells (Supplementary Fig. 7B), suggesting that loss of EPLIN may enhance medulloblastoma cell proliferation. We further analyzed two medulloblastoma datasets with available survival data using the same expression cutoff method in Fig. 3D, but found no significant differences in overall survival between high and low EPLIN expression groups (Supplementary Fig. 7C). This lack of association may be due to the molecular heterogeneity across medulloblastoma subgroups [45]. To explore this possibility, we re-analyzed data from the Hugene11t platform (GEO ID: GSE85217), which includes 763 primary medulloblastoma samples classified into Group 3, Group 4, WNT, and SHH subtypes. Within the SHH subgroup, lower EPLIN expression was significantly associated with poorer overall survival (*n* = 172, *p* = 0.018, Supplementary Fig. 7D), suggesting a potential subtype-specific role of EPLIN in medulloblastoma progression.

## Discussion

In this study, we aimed to identify novel compounds targeting previously unexplored proteins for the treatment of pediatric tumors, including neuroblastoma and medulloblastoma. To accomplish this, we screened a 1581-chemical library and identified FLIX5, a promising small molecule.

**Fig. 7:**
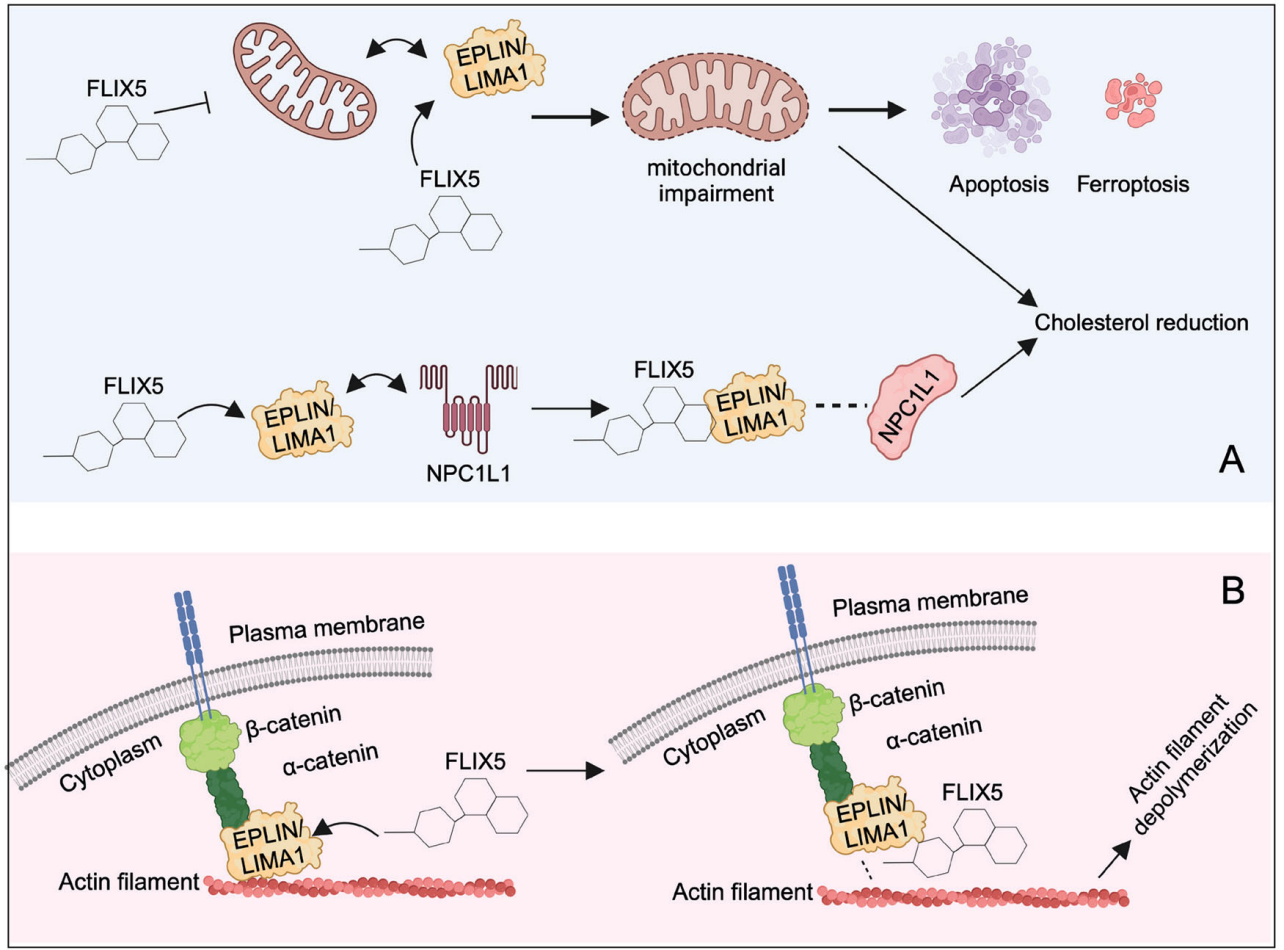
An illustration of the mechanism action of FLIX5. In summary, (**A**) FLIX5 has a multifaceted impact on cellular processes, including impairing the mitochondrial function, altering cholesterol levels, and inducing apoptosis and ferroptosis. **B** FLIX5 may disrupt the interaction between EPLIN and actin filaments, eventually causing actin filament depolymerization. Our study also suggests that reduced EPLIN expression in cancer acts as a double-edged sword. While low EPLIN levels can promote cancer initiation and progression, cancer cells with diminished EPLIN expression may become more reliant on the remaining protein to support essential cellular functions. Consequently, further disruption of EPLIN function or its interactions with other proteins may lead to increased toxicity in cancer cells compared to normal cells. The figure was created with Biorender.com.

Our findings demonstrate that FLIX5 effectively inhibits mitochondrial oxidative phosphorylation and significantly reduces both intracellular free and total cholesterol levels, showing a synergistic effect when combined with ezetimibe, an NPC1L1 inhibitor, as well as induces apoptosis (Fig. 7A). When combined with vincristine, FLIX5 consistently produced a stable synergistic effect in neuroblastoma cells and PDX organoids. Further scRNA-seq analysis of IMR-32 spheroids revealed that growth-arrested cells exhibit upregulation of fatty acid metabolism. This finding might explain the efficacy of FLIX5 in targeting spheroids, which harbor a substantial population of growth-arrested cancer cells.

Subsequent studies confirmed EPLIN as a target of FLIX5, potentially resulting in actin filament depolymerization (Fig. 7B). EPLIN is primarily expressed in human epithelial cells but is frequently downregulated in cancerous tissues. However, the full role of EPLIN in cellular processes remains incompletely understood. We have shown that reduced EPLIN expression has been linked to poor prognosis in neuroblastoma and the SHH subgroup of medulloblastoma. FLIX5 exhibits significantly higher toxicity toward neuroblastoma and medulloblastoma cells, with minimal toxicity in normal cells. Additionally, further reduction of EPLIN levels may enhance the sensitivity of cancer cells to FLIX5. Together, these findings suggest that decreased EPLIN expression in cancer cells acts as a double-edged sword. While low EPLIN levels can promote cancer initiation and progression, cancer cells with diminished EPLIN expression may become more reliant on the remaining protein to support essential cellular functions. Consequently, further disruption of EPLIN function or its interactions with other proteins may lead to increased toxicity in cancer cells compared to normal cells.

However, we acknowledge limitations in our study. Firstly, while we have demonstrated that EPLIN is a target of FLIX5, we cannot exclude the possibility that FLIX5 may also target other proteins within cancer cells. Given that EPLIN expression in cancer cells acts as a double-edged sword, there is a strong demand for the development of molecules with optimized chemical structures that specifically target EPLIN. Secondly, the full range of EPLIN functions remains unclear, particularly the cellular dependencies that arise from reduced EPLIN levels in cancer cells. Further investigation into the biological changes resulting from EPLIN reduction may uncover potential targets for pediatric cancer treatment.

In summary, the identification of EPLIN as a key player in cancer biology opens new avenues for investigating its role. Further research into the development of novel EPLIN-targeted therapies could provide promising treatment options for neuroblastoma, medulloblastoma, and other tumor types.

### Data sharing statement

The data supporting the findings of this study were included in the main and supplementary materials. Further information regarding this study is available from the corresponding author upon reasonable request.

## Supplementary information

Supplementary Figures and Figure legends

Reproducibility checklist

Supplementary Table 1

Supplementary Table 2

Supplementary Table 3

Supplementary Table 4

Supplementary Table 5

Supplementary Table 6

Supplementary Table 7

Original WBs

## Data Availability

Uncropped western blots are provided in the supplementary information. Additional data and materials that support the findings of the study can be found in the article and its supplementary material. The scRNA sequencing data generated were deposited with GEO accession numbers GSE275538. The code used for this analysis can be found: https://github.com/Summertree23/neuroblastoma.
