# Supplementary material for: Identification of a small molecule targeting EPLIN as a novel strategy for the treatment of pediatric neuroblastoma and medulloblastoma
Source: Cell Death Dis. 2025 Jul 23;16(1):554. doi: 10.1038/s41419-025-07876-7 (PMC12287531; doi:10.1038/s41419-025-07876-7)

Supplementary Figure legends

Supplementary Figure 1. Screening of chemical libraries identifies toxic agents for neuroblastoma cells, regardless of MYCN status, in both 2D and 3D conditions

A. Dot plots showing the cell viability of 1581 compounds at 10μM. Black dots represent compounds with <20% cytotoxicity, which were excluded. Red dots represent compounds with >20% cytotoxicity, which in total 484 compounds, were retained for further testing.

B. Cell viability of five compounds - FLIX1 (NSC105827), FLIX2 (NSC607097), FLIX3 (NSC354844), FLIX4 (NSC330770), and FLIX5 (NSC328403) - was assessed. These compounds were identified based on their cytotoxicity being >50% against 5 neuroblastoma spheroids, with or without MYCN overexpression, at 1μM after 72 hours of treatment.

C. Dose-response of FLIX5 on SH-SY5Y spheroids. One representative experiment with 3 technical replicates is shown (mean ± SD).

D. Dose-response of FLIX5 on hRPE1 spheroids at concentrations up to 5000nM. One representative experiment with three technical replicates is shown (mean ± SD).

E. Acquired images of SK-N-AS spheroids after exposure to FLIX5 for 72 hours at the indicated concentrations. Circles outlined in yellow delineate the spheroid core and the remaining part of the spheroid. The diameter of the core is utilized for the calculation of volume.

F. Quantification of calculated spheroid volume of SK-N-AS exposed to FLIX5 in (E). The volume of the spheroids was calculated by the equation: $V=4/3\times\pi\times r^3$ (r=radius). Fold change obtained by normalized the volume to the condition of DMSO treatment. One representative experiment with 3 technical replicates is shown (mean ± SD, ns. $p>0.05$; ***$p < 0.001$; t-test).

Supplementary Figure 2. FLIX5 targets EPLIN in neuroblastoma cells

A. Network result of top 50 proteins with significant changes in solubility in cells treated with FLIX5. Red and green rounds are the top 50 with significantly increased and decreased solubility respectively in treated cells. Red rounds in A show the proteins enriched in RNA binding.

B. The Gibbs free energy using the relationship $\Delta G = -RT \ln (K_{eq})$, where R is the gas constant, T is the temperature and K_{eq} is the equilibrium constant. Statistical analysis, such as error estimation and confidence intervals, was performed to evaluate the reliability of the calculated binding free energies. The entire 500ns simulation depicted a favorable binding free energy for FLIX5 toward the EPLIN protein is VDWAALS (Van der Waals forces) and GGAS (Guanine-Guanine Adenine-Adenine Stacking).

C. Left: the CETSA experiment was further validated in samples treated with either DMSO or FLIX5 (0.5μM) for one hour using CHP-212 cells. The temperature tested ranged from 40°C to 60°C, with intervals of 5°C. Right: quantification of band densities in the Western blots at the indicated temperatures. The CETSA experiment was repeated at least three times independently.

Supplementary Figure 3. Reduction in EPLIN expression is associated with poorer overall survival

A. The overall survival rates of neuroblastoma patients with high and low EPLIN gene expression (the first quartile vs. the last quartile) were examined using Kaplan-Meier survival curves in Asgharzadeh-249 which include 249 neuroblastoma samples. Statistical analyses were conducted using the log-rank test, with data sourced from publicly available patient cohorts on the R2 microarray analysis and visualization platform.

Supplementary Figure 4. FLIX5 impairs mitochondrial function and increases the dependency of neuroblastoma cells on lipid metabolism.

A. Intracellular ATP levels in CHP-212 cells exposed to the indicated concentrations of FLIX5 for 24 and 48 hours. Data from one representative experiment with 6 technical replicates are shown (mean ± SD). Statistical significance was calculated using an unpaired two-tailed t-test ($p < 0.05$ is considered significant).
B. Intracellular ATP levels in DAOY parental and EPLIN KO cells. Data from one representative experiment with 4 technical replicates are shown (mean ± SD). Statistical significance was calculated using an unpaired two-tailed t-test ($p < 0.05$ is considered significant).
C. The summarized list of proteins directly interacting with EPLIN.
D. Immunochemistry staining of IMR-32 spheroids using the proliferation marker Ki67 and the quiescent marker P27.
E. GSEA enrichment profile of the significantly upregulated hallmark pathways of protein secretion (p-value = 0.0014, adjusted p-value = 0.0053) and mTORC1 signaling (p-value = 0.0012, adjusted p-value = 0.0053) in Group 3.

Supplementary Figure 5. Up- and down- regulated proteins in FLIX5 exposed cells

A. The list of significantly upregulated genes in FLIX5-treated cells (48 hours). From this analysis, we identified 50 proteins significantly upregulated (fold change FLIX5/DMSO > 1.5, p-value < 0.05) in FLIX5-treated cells.
B. The list of significantly down-regulated genes in FLIX5-treated cells (48 hours). From this analysis, we identified 41 proteins significantly downregulated (fold change FLIX5/DMSO

< 0.85, p-value ≤ 0.05) in FLIX5-treated cells.

C. Network nodes of the top 50 upregulated proteins in FLIX5-treated samples. The protein list was obtained from the list in A.
D. Network nodes of the top 41 downregulated proteins in FLIX5-treated samples. The protein list was obtained from the list in B.
E. Immunoblots were conducted to detect changes in the expression of p-4EBP1 and LC3 in cells treated with either DMSO or FLIX5 for 24 or 48 hours. Actin is the loading control.
F. Quantified Caspase 3/7 activity by GFP signalling (%) in CHP-212 cells treated with DMSO (3% - green) and FLIX5 for 48 hours (11% - pink).

Supplementary Figure 6. FLIX5 works synergistically with vincristine in the treatment of neuroblastoma.

A. The combination effect of FLIX5 with vincristine or vinorelbine, and with docetaxel or paclitaxel in CHP-212 cells were analyzed. Data were processed using the MacSynergy™ II. A synergy score of >1, =1, or <1 indicates that the drugs are synergistic, additive, or antagonistic, respectively.
B. The combination effect of FLIX5 with vincristine, vinorelbine, or docetaxel in IMR-32 cells was analyzed. Data was processed using the MacSynergy™ II as previously.
C. The top significantly upregulated GO molecular function pathways in the specified treatment groups. A complete list of the altered pathways with their significance is provided in Supplementary Table 7.
D. Network result of genes involved in heterodimerization activity and endopeptidase regulation pathways.
E. The top significantly down-regulated Reactome pathways in the indicated treatment groups. The list of altered pathways can be found in Supplementary Table 7.

F-I: Three-dimensional model analyzing the interactions between FLIX5 and vincristine or vinorelbine in LU-NB-1 and LU-NB-2. Data were analyzed using the MacSynergy™ II excel spreadsheet and ZIP synergy scoring method, as previously described.

Supplementary Figure 7. Role of EPLIN in Medulloblastoma.

A. Expression levels of EPLIN in DAOY parental cells and EPLIN knockout clones. Actin was used as a loading control.
B. Comparison of cell growth between DAOY parental and KO clones, normalized to day 0.
C. Kaplan-Meier survival analysis of medulloblastoma patients with high vs. low EPLIN expression (top quartile vs. bottom quartile) in the Williamson-331 and Cavalli-763 cohorts, which include 331 and 763 patient samples, respectively. Statistical significance was assessed using the log-rank test. Data were obtained from the publicly available R2

microarray analysis and visualization platform.

D. Re-analysis of data from the Cavalli-763 Hugene11t cohort (GEO ID: GSE85217), stratified into Group 3, Group 4, WNT, and SHH molecular subtypes. In the SHH subgroup, lower EPLIN expression was significantly associated with reduced overall survival (n = 172, p = 0.018).

Supplementary Figure 1

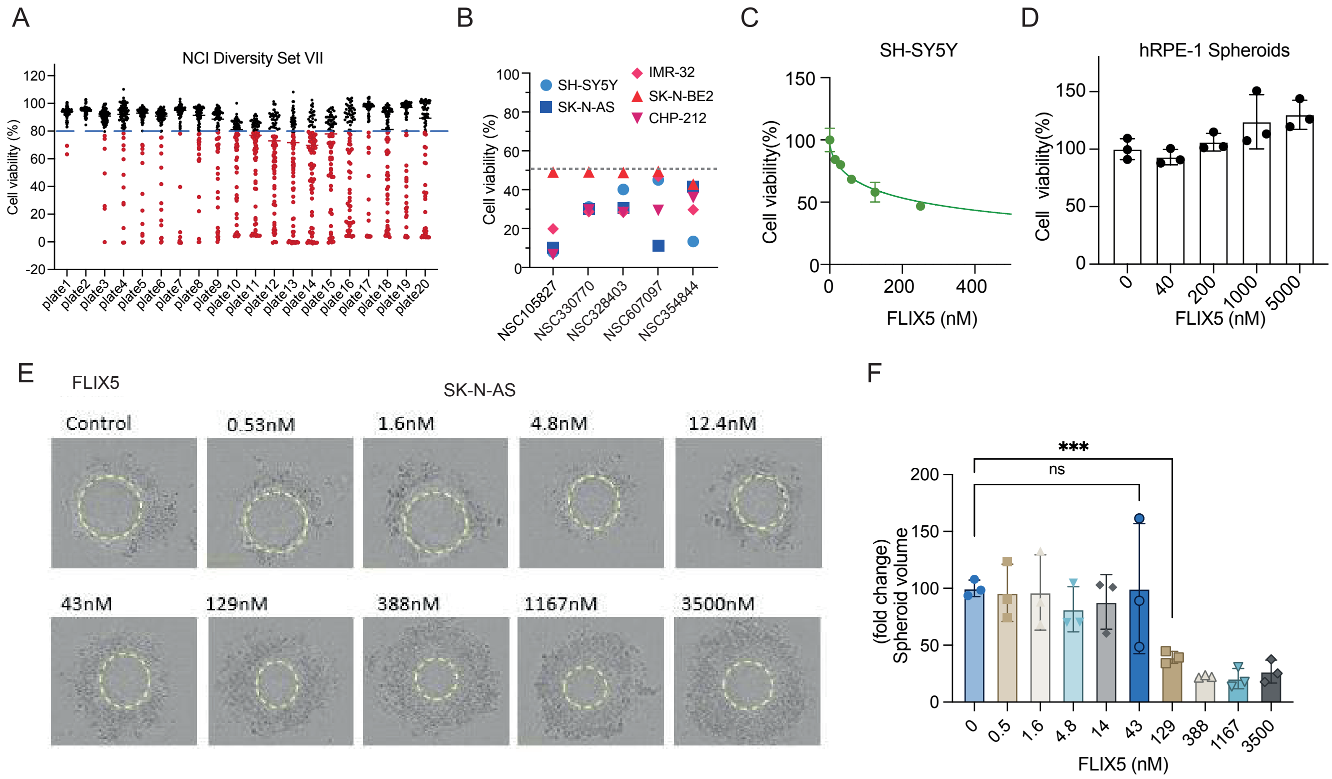

Supplementary Figure 2

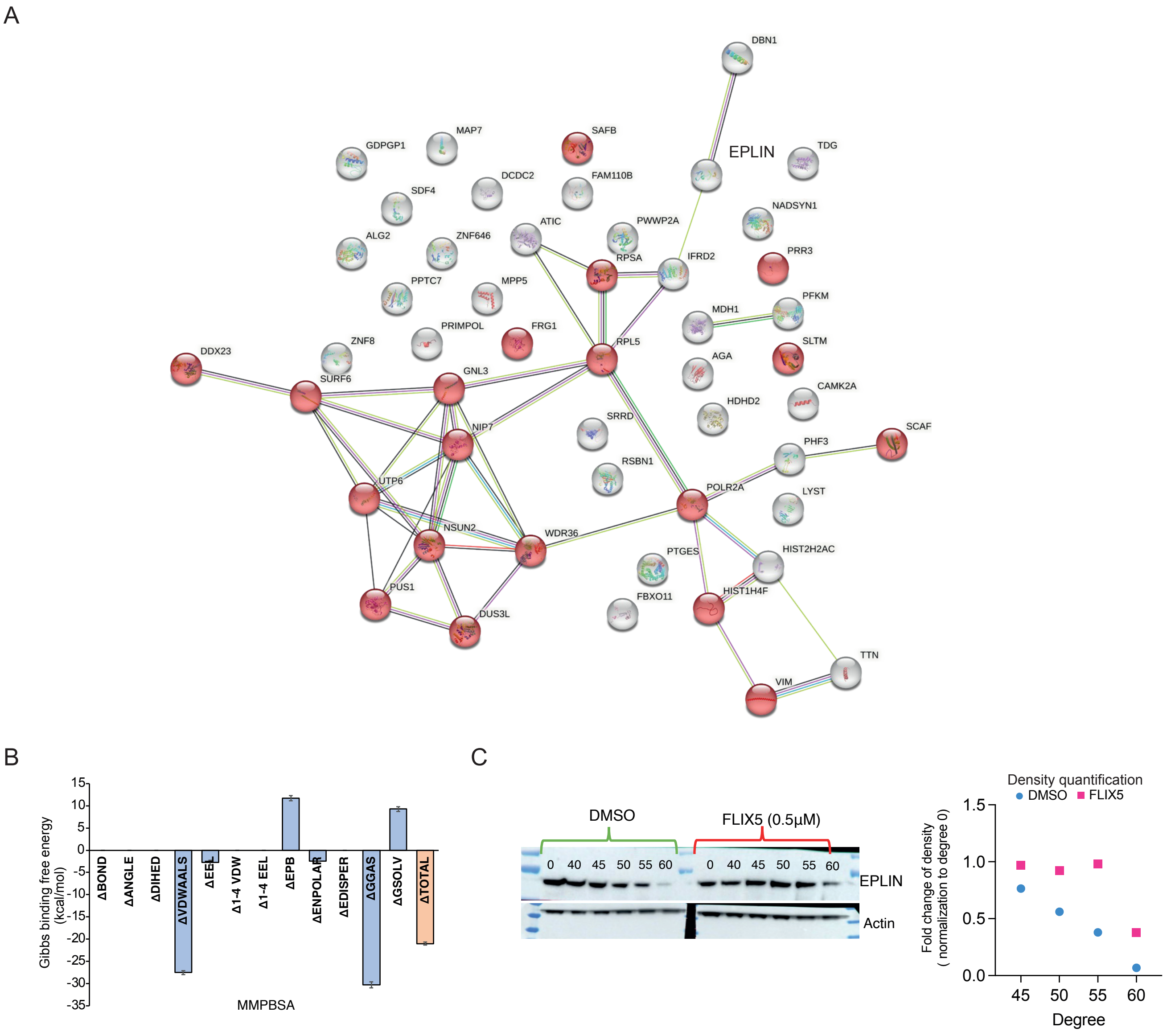

Supplementary Figure 3

A

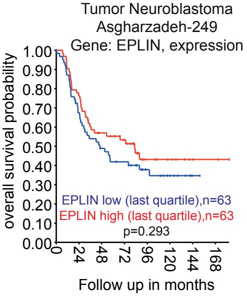

Supplementary Figure 4

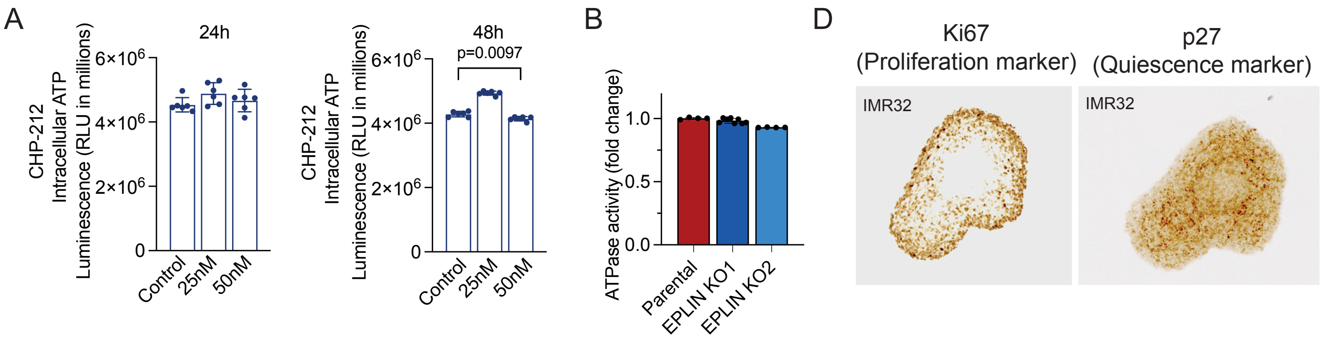

C

**Cytoskeleton and regulation**	
Actin, cytoplasmic 1	actin
Actin, alpha cardiac muscle 1	actin
Tubulin beta-4B chain	tubulin
Ezrin	an intermediate between the plasma membrane and the actin cytoskeleton
WASH complex subunit FAM21	induce actin polymerization,
myosin-Vb	actin binding
Drebrin-like protein	actin cytoskeleton,
Cofilin-1	Cofilin-1
**Protein regulation**	
Heat shock protein HSP 90-alpha	chaperone
Slc5a4b,Solute carrier family 15	transporter
Elongation factor 1-delta	translation
Eukaryotic initiation factor 4A-II	translation
40S ribosomal protein S10	translation
GRB2-associated-binding protein 1	cellular growth response
**Metabolism**	
Niemann-Pick C1-like protein 1	Cholesterol Transporter
Armadillo repeat-containing protein 4	steroidogenesis
AP-2 complex	polyphosphoinositide-containing lipids
Histamine N-methyltransferase	metabolize histamine
**Adherens junctions**	
Catenin delta-1	adherens junctions
Claudin-3	tight junctions
Cadherin-17	cell adhesion proteins
**Others**	
Interferon-induced transmembrane protein 2	antiproliferative action of IFN-gamma
Interferon-induced transmembrane protein 3	antiproliferative action of IFN-gamma
Dynein light chain 2	mitotic spindle formation.
Calmodulin-like protein	unconventional myosin-10 (MYO10)
Transgelin-2	calcium ion binding.

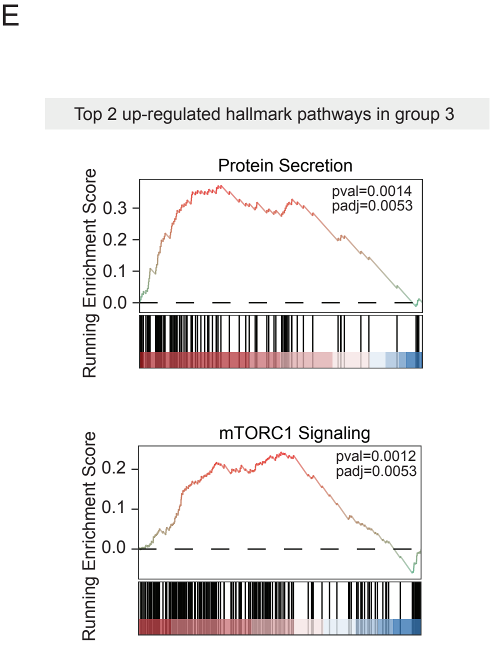

Supplementary Figure 5

A

Significant up-regulated genes in FLIX5 treated cells (48h)

Gene Symbol	Description	Fold change (Cpd5/DMSO)	p value
KHDC1L	Putative KHDC1-like protein	1,76	0,00
METTL25B	Methyltransferase-like protein 25B	1,63	0,00
RGS4	Regulator of G-protein signaling 4	1,77	0,00
TCHP	Trichoplein keratin filament-binding protein	1,66	0,00
GDF15	Growth/differentiation factor 15 O	2,05	0,00
GIGYF1	GRB10-interacting GYF protein 1	1,67	0,00
LONRF3	LON peptidase N-terminal domain and RING finger protein 3	1,65	0,00
DNAJC19	Isoform of Q96DA6, Mitochondrial import inner membrane translocase subunit TIM14	1,67	0,00
HABP2	Hyaluronan-binding protein 2	1,74	0,00
ZCCHC14	Isoform of Q8WYQ9, Zinc finger CCHC domain-containing protein 14	1,61	0,00
LRPPRC	Isoform of P42704, Leucine-rich PPR motif-containing protein, mitochondrial	1,78	0,00
CCDC18	Isoform of Q5T9S5, Coiled-coil domain-containing protein 18 (Fragment)	1,62	0,00
DNAH10	Isoform of Q8IVF4, Dynein axonemal heavy chain 10	1,66	0,00
ALG9	Alpha-1,2-mannosyltransferase ALG9	1,95	0,00
RMND5A	E3 ubiquitin-protein transferase RMND5A	1,60	0,01
TSNAXIP1	Isoform of Q2TAA8, Translin-associated factor X-interacting protein 1	1,81	0,01
THBS2	Thrombospondin-2	1,76	0,01
PRCP	Isoform of P42785, Lysosomal Pro-X carboxypeptidase	1,80	0,01
PLCB2	1-phosphatidylinositol 4,5-bisphosphate phosphodiesterase beta-2	1,63	0,01
DNAH14	Isoform of Q0VDD8, Dynein axonemal heavy chain 14	1,79	0,01
MAPKBP1	Mitogen-activated protein kinase-binding protein 1	1,72	0,01
TIAM1	Rho guanine nucleotide exchange factor TIAM1	2,27	0,01
HNRNPD	Isoform of Q14103, Heterogeneous nuclear ribonucleoprotein D0	1,67	0,01
MYH7	Myosin-7	1,68	0,01
KCNQ5	Potassium voltage-gated channel subfamily KQT member 5	1,92	0,01
CLHC1	Clathrin heavy chain linker domain-containing protein 1	2,02	0,01
STAM	Isoform of Q92783, Signal transducing adapter molecule 1	2,10	0,01
MTDH	Isoform of Q86UE4, Protein LYRIC	1,98	0,01
KIF2B	Kinesin-like protein KIF2B	1,85	0,01
RAB20	Ras-related protein Rab-20	1,62	0,01
ABCB11	Bile salt export pump	1,99	0,01
SAE1	Isoform of Q9UBE0, SUMO-activating enzyme subunit 1	1,65	0,01
ZNF573	Zinc finger protein 573	1,82	0,01
TENM3	Teneurin-3	1,64	0,02
FAM83H	Isoform of Q6ZRV2, Protein FAM83H	2,00	0,02
EIF3B	Isoform of P55884, Eukaryotic translation initiation factor 3 subunit B	1,60	0,02
ZNF704	Zinc finger protein 704	1,72	0,02
ZNF101	Zinc finger protein 101	2,05	0,03
HBA1	Isoform of P69905, Alpha-globin	1,61	0,03
KIF1B	Isoform of O60333, Isoform 2 of Kinesin-like protein KIF1B	1,63	0,03
CMYA5	Cardiomyopathy-associated protein 5	2,81	0,04

B

Significant down-regulated genes in FLIX5 treated cells (48h)

Gene Symbol	Description	Fold change Cpd5/DMSO	p value
MAP2	Microtubule-associated protein 2	0,79	0,00
FAM107B	Protein FAM107B	0,78	0,00
PGM5	Phosphoglucomutase-like protein 5	0,80	0,00
COL6A6	Collagen alpha-6(VI) chain	0,80	0,00
FADS2	Acyl-CoA 6-desaturase	0,70	0,00
GDI2	Rab GDP dissociation inhibitor beta	0,82	0,00
AARS1	Alanine--tRNA ligase, cytoplasmic	0,72	0,00
TUBA1C	Isoform of Q9BQE3, Tubulin alpha chain	0,83	0,00
SPARC	SPARC	0,82	0,00
TGM2	Protein-glutamine gamma-glutamyltransferase 2	0,81	0,00
MAP9	Microtubule-associated protein 9	0,81	0,00
PODXL	Podocalyxin	0,79	0,00
ANXA1	Annexin A1	0,83	0,00
KANK4	KN motif and ankyrin repeat domain-containing protein 4	0,80	0,00
CTSS	Cathepsin S	0,84	0,00
VAMP5	Vesicle-associated membrane protein 5	0,72	0,00
CD44	CD44 antigen	0,82	0,00
AARSD1	Alanyl-tRNA editing protein Aarsd1	0,82	0,00
PRR15	Proline-rich protein 15	0,79	0,00
SDC3	Syndecan-3	0,73	0,00
ACBD7	Acyl-CoA-binding domain-containing protein 7	0,84	0,00
ADI1	Acireductone dioxygenase	0,76	0,00
GNG11	Guanine nucleotide-binding protein G(I)/G(S)/G(O) subunit gamma-11	0,67	0,00
FHL2	Four and a half LIM domains protein 2	0,83	0,00
MIF	Macrophage migration inhibitory factor	0,83	0,00
ALDH1A1	Aldehyde dehydrogenase 1A1	0,81	0,00
TUBB2A	Tubulin beta-2A chain	0,79	0,00
HDDC2	5'-deoxynucleotidase HDDC2	0,83	0,00
CALB2	Isoform of P22676, Calretinin (Fragment)	0,75	0,01
RMI2	Isoform of Q96E14, RecQ-mediated genome instability protein 2	0,83	0,01
MSH5	Isoform of O43196, MutS protein homolog 5 (Fragment)	0,77	0,02
CREB1	Isoform of P16220, Isoform 2 of Cyclic AMP-responsive element-binding protein 1	0,82	0,02
MT2A	Metallothionein-2	0,83	0,02
SPTA1	Spectrin alpha chain, erythrocytic 1	0,83	0,02
CTNNBIP1	Beta-catenin-interacting protein 1	0,80	0,03
METAP2	Methionine aminopeptidase 2	0,49	0,03
TUBB8	Tubulin beta-8 chain	0,81	0,03
ANKRD20A 1	Isoform of Q5TYW2, Ankyrin repeat domain-containing protein 20A1	0,72	0,03
CKS1B	Cyclin-dependent kinases regulatory subunit 1	0,83	0,04
POLR3A	Isoform of O14802, DNA-directed RNA polymerase (Fragment)	0,81	0,05
SERPINB8	Serpin B8	0,83	0,05

Supplementary Figure 5

C

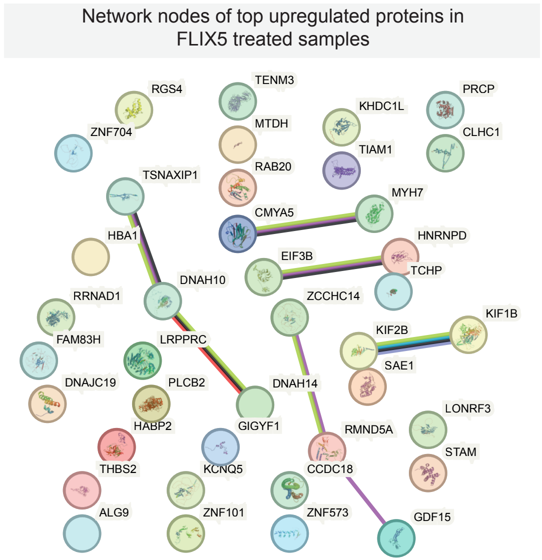

D

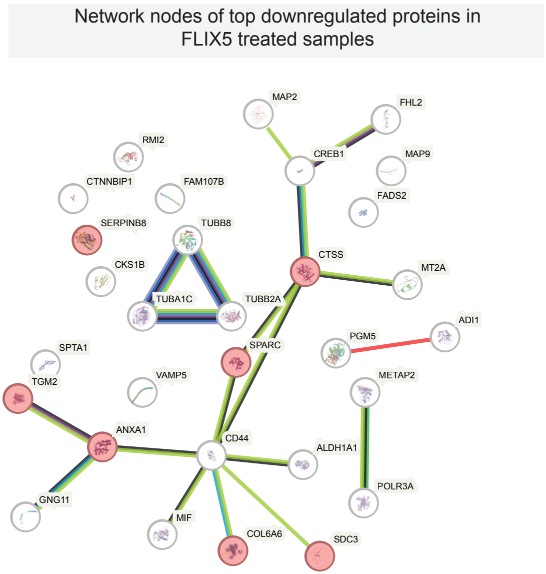

E

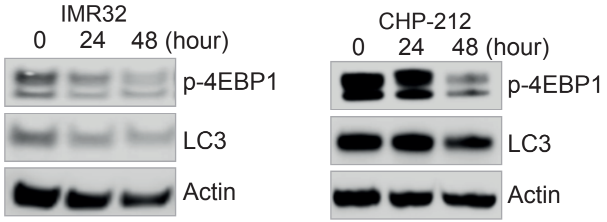

F

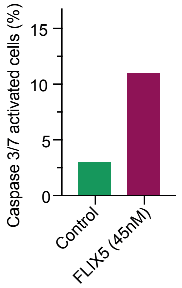

Supplementary Figure 6

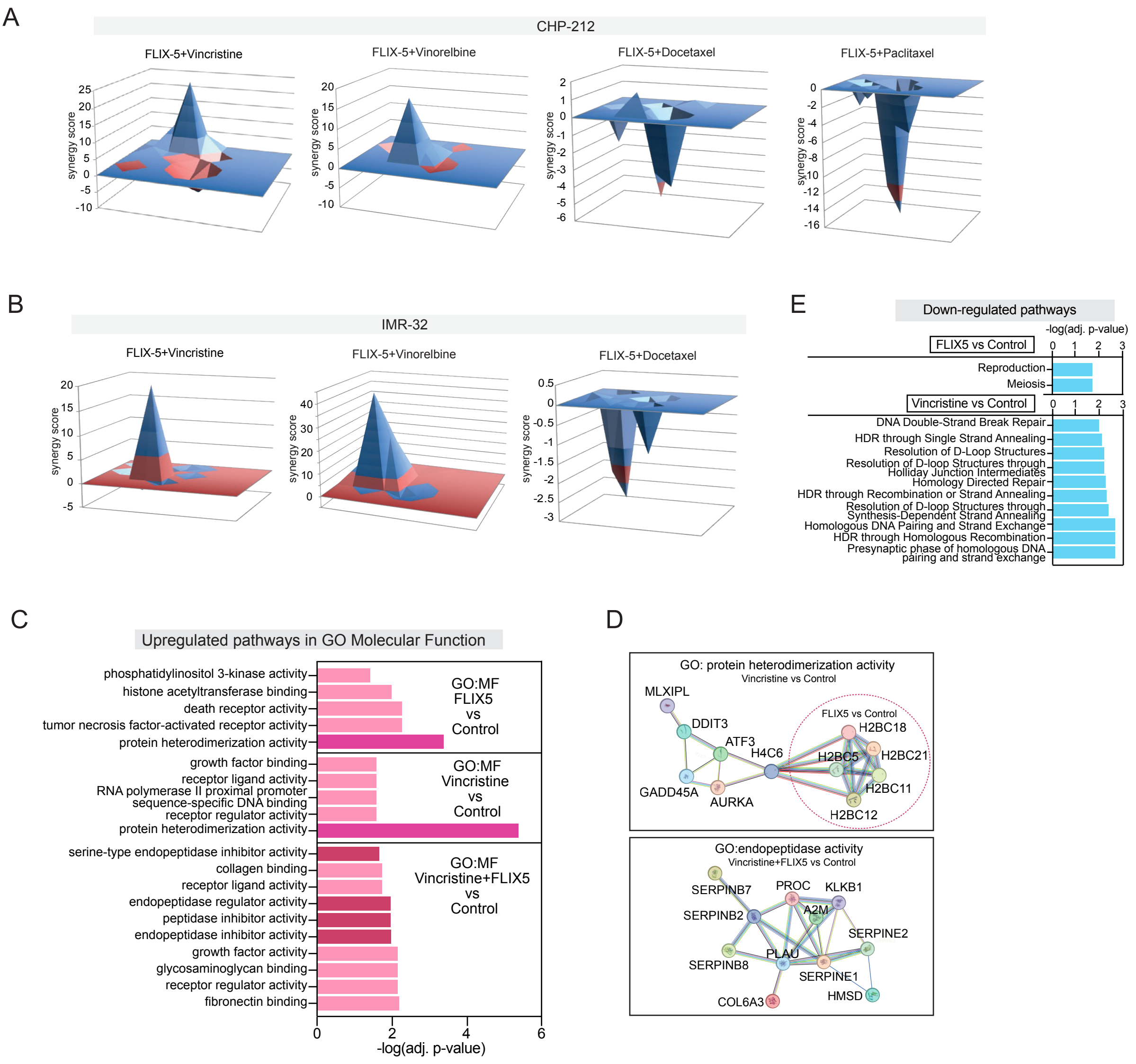

Supplementary Figure 6

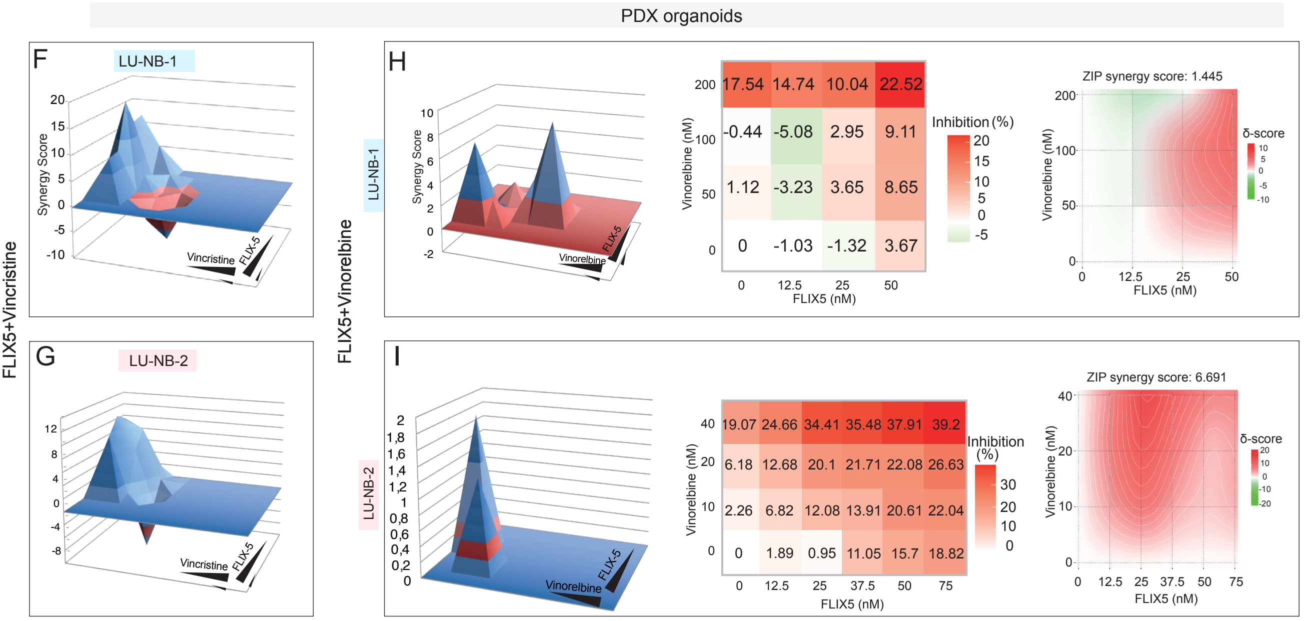

Supplementary Figure 7

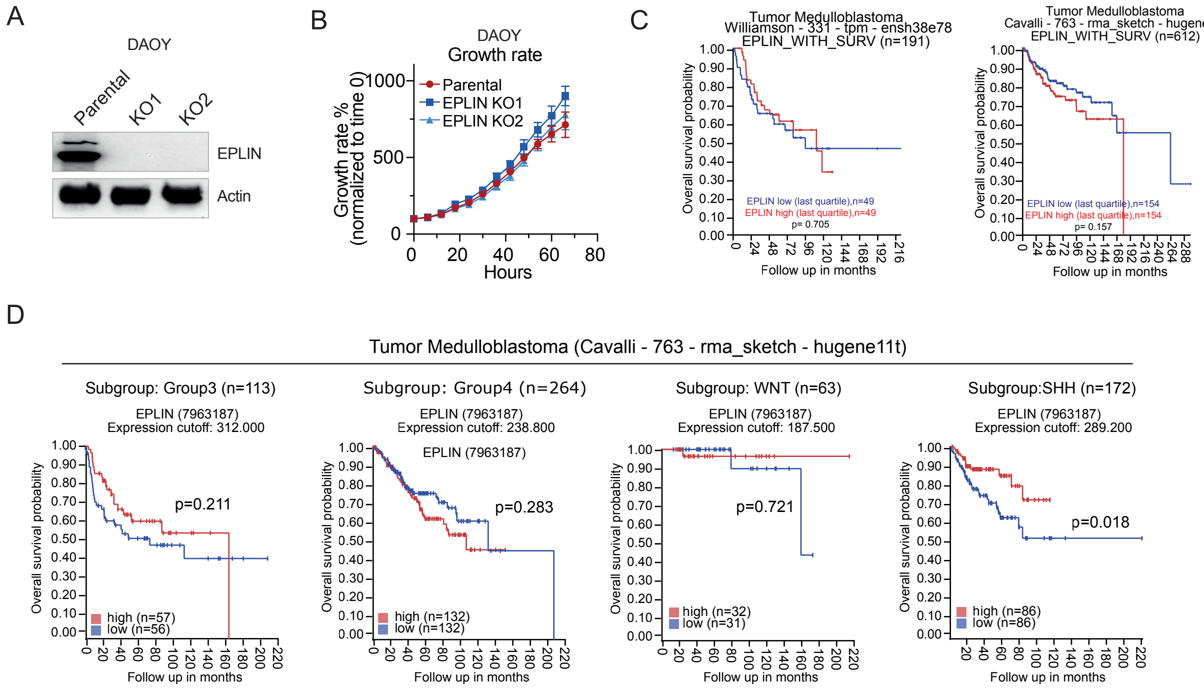

Supplement: Supplementary file 1 — Supplementary Figures and Figure legends [file 41419_2025_7876_MOESM1_ESM.pdf]
