# Supplementary material for: Identification of a small molecule targeting EPLIN as a novel strategy for the treatment of pediatric neuroblastoma and medulloblastoma
Source: Cell Death Dis. 2025 Jul 23;16(1):554. doi: 10.1038/s41419-025-07876-7 (PMC12287531; doi:10.1038/s41419-025-07876-7)
Supplement: Supplementary file 2 — Reproducibility checklist [file 41419_2025_7876_MOESM2_ESM.pdf]

| Reagents | | |
|---|---|---|
| **Name** | **Company** | **Product/Catalog number** |
| Ham’s F-12 Nutrient Mix | Thermo Fisher Scientific, Waltham, MA,USA | #11765054 |
| EMEM | ATCC | #30-2003 |
| Hanks´balanced salt solution (HBSS) | Thermo Fisher Scientific, Waltham, MA,USA | #14175-053 |
| Fetal Bovine Serum (FBS) | Thermo Fisher Scientific, Waltham, MA,USA | #10270-106 |
| Penicillin-Streptomycin | Thermo Fisher Scientific, Waltham, MA,USA | # 15140-122 |
| Trypsin-EDTA (0,25%), phenol red | Thermo Fisher Scientific, Waltham, MA,USA | #25200056 |
| DMEDM (1x) + GlutMAX (Serum free medium) | Gibco, Thermo Fisher Scientific, Waltham, MA,USA | #21885-025 |
| Neurobasal – A medium | Thermo Fisher Scientific, Waltham, MA,USA | #10888022 |
| B27-A | Thermo Fisher Scientific, Waltham, MA,USA | #12587010 |
| bFGF | Thermo Fisher Scientific, Waltham, MA,USA | # 100-18B |
| L-glutamine (100x) | Thermo Fisher Scientific, Waltham, MA,USA | #25030081 |
| EGF | Sigma-Aldrich, St Lousie, MO, USA | #E9644 |
| Dulbecco’s modified Eagle’s low glucose medium | Thermo Fisher Scientific, Waltham, MA,USA | #21885108 |
| Ham's F-12 Nutrient Mix, GlutaMAX™ Supplement | Thermo Fisher Scientific, Waltham, MA,USA | #31765027 |
| B27 without vitamin A | Thermo Fisher Scientific, Waltham, MA,USA | #12587010 |
| Fibroblast growth factor | Peprotech | #100-18B |
| epidermal growth factor | Peprotech | #AF-100-15 |
| Accutase | Sigma-Aldrich, St Lousie, MO, USA | #A6964 |
| Resazurin | Sigma-Aldrich, St Lousie, MO, USA | #R12204 |
| Trypan Blue stain (0,4%) | Thermo Fisher Scientific, Waltham, MA,USA | #T10282 |
| Dimethyl sulfoxide (DMSO) | Sigma-Aldrich, St Louis, MO, USA | #D8418 |
| BSA | Roche Diagnostics GmbH, DE | #10735094001 |
| RIPA lysis and extraction buffer | Thermo Fisher Scientific, Waltham, MA,USA | #89901 |

| | | |
|---|---|---|
| Pierce™ BCA Protein Assay Kits | Thermo Fisher Scientific, Waltham, MA,USA | # 23227 |
| PBS tablets | Thermo Fisher Scientific, Waltham, MA,USA | #18912-014 |
| Tween® 20 | Sigma-Aldrich, St Louis, MO, USA | #P1379 |
| Skim Milk Powder | Millipore, Germany | # 1.15363.0500 |
| SuperSignal™ West Pico PLUS Chemiluminescent Substrate | Thermo Fisher Scientific, Waltham, MA,USA | #34577 |
| Protease inhibitor cocktail | Sigma-Aldrich, St Louis, MO, USA | #P8340 |
| Protease and phosphatase inhibitor cocktail | Sigma-Aldrich, St Louis, MO, USA | #PPC1010 |
| MES SDS Running Buffer (20x) | Invitrogen, Thermo Fisher Scientific, Waltham, MA,USA | #NP0002 |
| MOPS SDS Running Buffer (20x) | Invitrogen, Thermo Fisher Scientific, Waltham, MA,USA | #NP0001 |
| BCA-assay | Invitrogen, Thermo Fisher Scientific, Waltham, MA,USA | # 23227 |
| Caspase3/7 Cell event kit | Invitrogen, Thermo Fisher Scientific, Waltham, MA,USA | #C10723 |
| M30 Apoptosense® ELISA | VLVbio, Sweden | #10011 |
| Seahorse Cell Mito Stress Test Kit | Agilent Technologies | #103015-100 |
| CellTiter-Glo 2.0 | Promega, Madison, WI, USA | #G9243 |
| **Materials** | | |
| **Name** | **Company** | **Product/Catalog number** |
| 96-well ultra-low attachment plates | Corning, NY, USA | #7007 |
| 1.5mm NuPAGE 12% Bis-Tris mini protein gels | Invitrogen, Thermo Fisher Scientific, Waltham, MA,USA | #NP0355BOX |
| iBlot™ 2 Transfer Stacks, nitrocellulose, regular size | Invitrogen, Thermo Fisher Scientific, Waltham, MA,USA | #IB23001 |
| iBlot® 7-minute blotting system | Invitrogen, Thermo Fisher Scientific, Waltham, MA,USA | #IB21001 |
| 96-well white surface plates | Thermo Fisher | #136101 |
| **Cells** | | |
| **Cell line** | **Company** | **Product/Catalog number** |
| SK-N-AS | ATCC, Manassas, VA, USA | Cat. #CRL-2137 |
| SH-SY5Y | ATCC, Manassas, VA, USA | Cat. #CRL-2266 |

| | | |
|---|---|---|
| SK-N-SH | ATCC, Manassas, VA, USA | Cat. #HTB-11 |
| CHP-212 | ATCC, Manassas, VA, USA | Cat. #CRL-2137 |
| IMR-32 | ATCC, Manassas, VA, USA | Cat. #CRL-127 |
| SK-N-BE (2) | ATCC, Manassas, VA, USA | Cat. #CRL-2271 |
| hRPE1 | ATCC, Manassas, VA, USA | Cat. #CRL-4000 |
| HDFa | ATCC, Manassas, VA, USA | Cat. # PCS-201-012 |
| D283 | ATCC, Manassas, VA, USA | Cat. #HTB-185 |
| Daoy | ATCC, Manassas, VA, USA | Cat. #HTB-186 |
| **Chemichals** | | |
| **Chemichal** | **Company** | **Product number** |
| Etoposide | Selleckchem, Germany | #S1225 |
| Staurosporine | MedChemExpress, NJ, USA | #HY-15141 |
| Ezetemibe | MedChemExpress, NJ, USA | #HY-15141 |
| Vincristine | NCI Diversity set VII | |
| Vinorelbin | NCI Diversity set VII | |
| Paclitaxel | NCI Diversity set VII | |
| Docetaxel | NCI Diversity set VII | |

| **Antibodies** | | | | |
|---|---|---|---|---|
| **Primary Antibody** | **Source** | **Dilution/Producer** | **Product number** | **Size (kDa)** |
| Actin | mouse | 1:1000, Santa Cruz Biotechnologies, Dallas, TX, USA | #sc-47778 | 42 kDa |
| MYCN | mouse | 1:1000, Abcam, Cambridge, UK | #16898 | 60kDa |
| p-4EBP1 | rabbit | 1:1000, Cell Signaling, Danvers, MA, USA, | #2855 | 20kDa |
| LIMA1 (EPLIN) | rabbit- | 1:1000, Sigma-Aldrich, St Louis, MO, USA | #HPA052645 | 100 kDa |
| SRRD | rabbit | 1:1000, Novus Biological, UK | #NBP1-70715 | 38kDa |

| | | | | |
|---|---|---|---|---|
| Cleaved PARP | rabbit | 1:1000, Cell Signaling Technology, Danvers, MA, USA | #5625 | 89kDa |
| Cleaved Caspase-3 | rabbit | 1:500, Cell Signaling Technology, Danvers, MA, USA | #9661S | 17/19v kDa |
| LC3A/B | rabbit | 1:1000, Cell Signaling Technology, Danvers, MA, USA | #12741S | 15/17 kDa |
| p53 | rabbit | 1:1000, Invitrogen, Massachusetts, USA | #PA5-27822 | 53 kDa |
| Phospho-Histone H2A.X | rabbit | 1:1000, Cell Signaling Technology, Danvers, MA, USA | #2577 | 15kDa |
| **Secondary Antibody** | **Source** | **Dilution/Producer** | **Product number** | |
| Goat anti-**Mouse** IgG (H+L) Secondary Antibody, HRP | Goat | 1:5000, Invitrogen, MA, USA | #31430 | |
| Goat anti-**Rabbit** IgG (H+L) Secondary Antibody, HRP | Goat | 1:5000, Invitrogen, MA, USA | #31460 | |
