# Supplementary material for: Identification of a small molecule targeting EPLIN as a novel strategy for the treatment of pediatric neuroblastoma and medulloblastoma
Source: Cell Death Dis. 2025 Jul 23;16(1):554. doi: 10.1038/s41419-025-07876-7 (PMC12287531; doi:10.1038/s41419-025-07876-7)

Figure 2J

SRRD

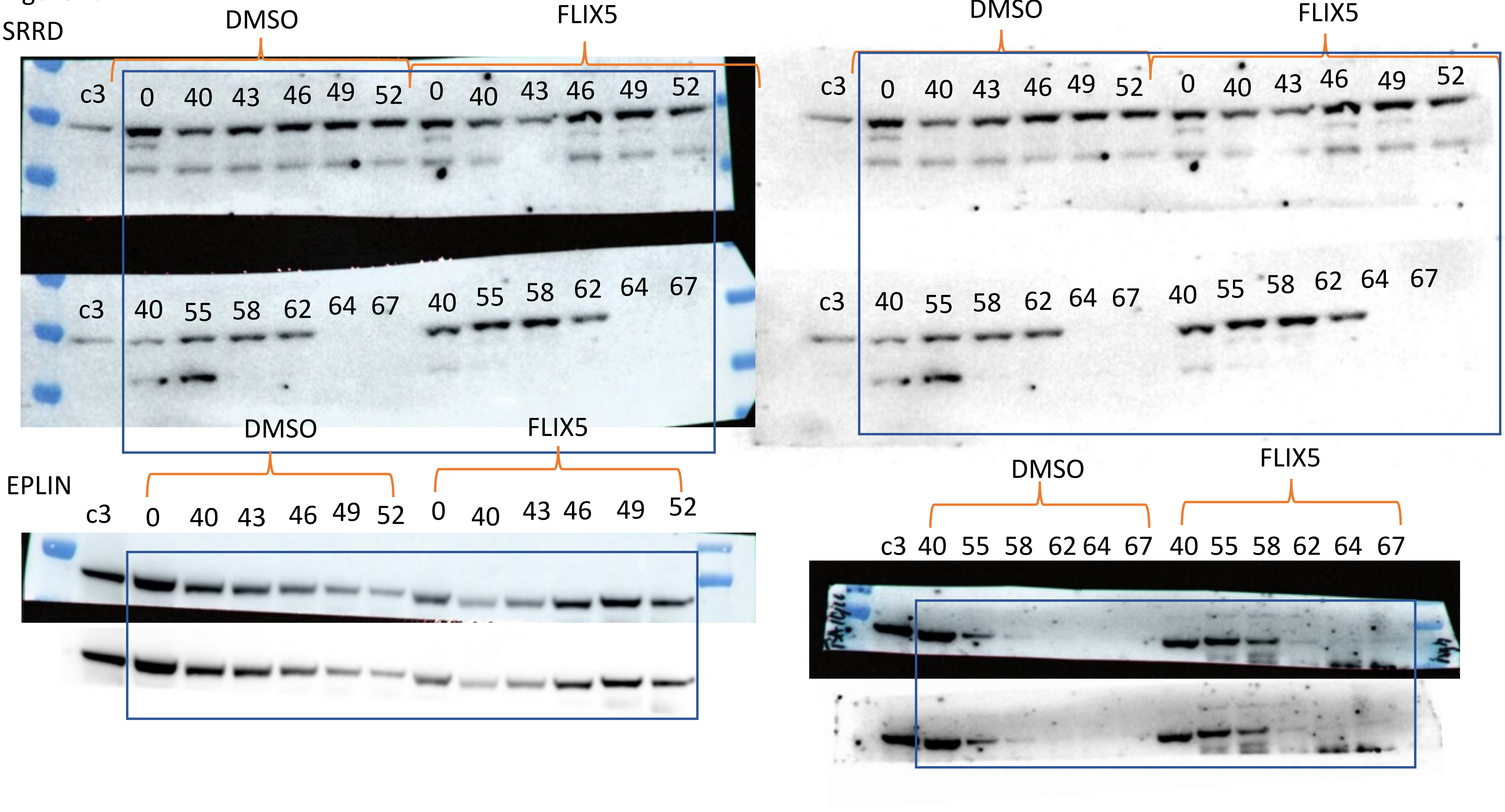

Figure 2K

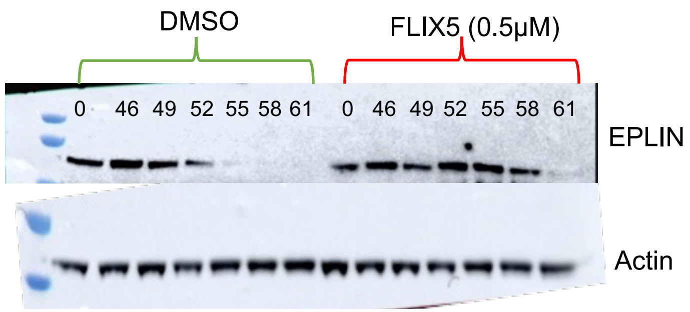

Figure 3D

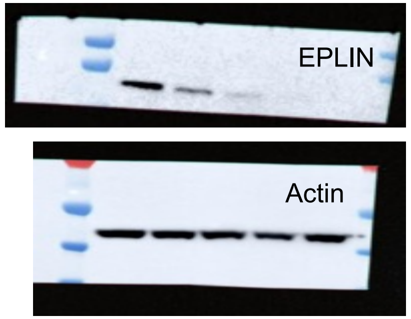

Figure 3F

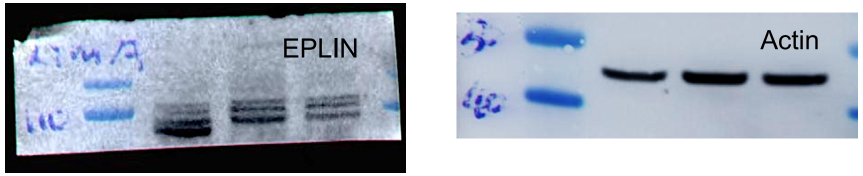

Figure 5E

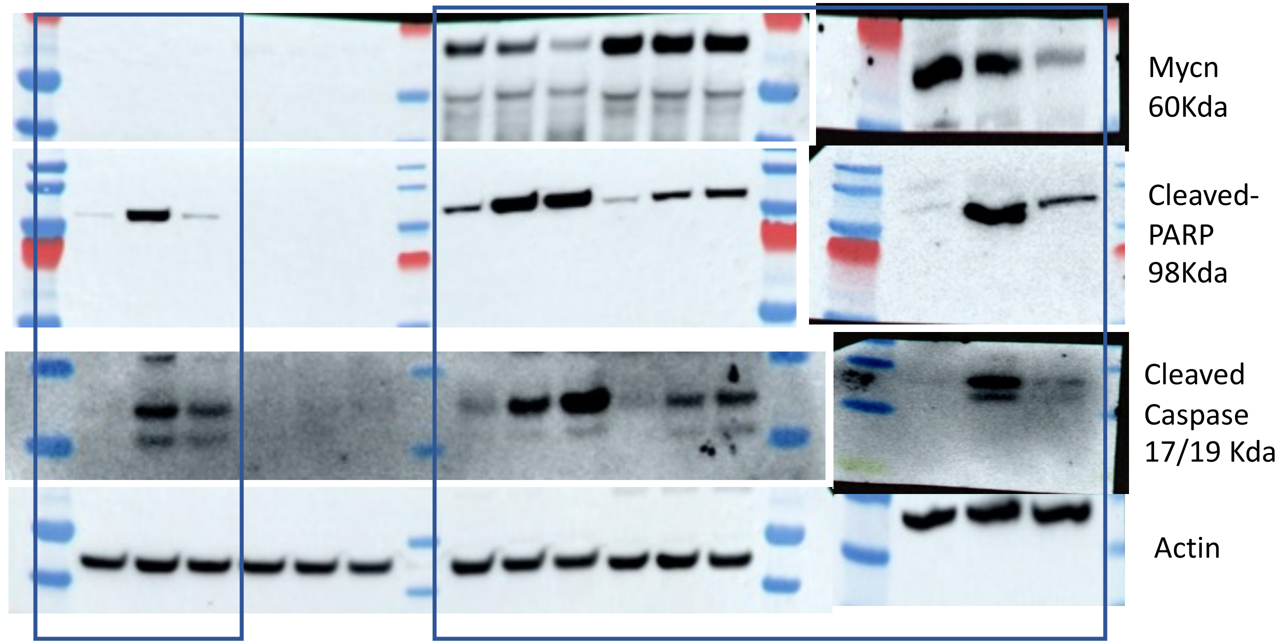

Figure 5G

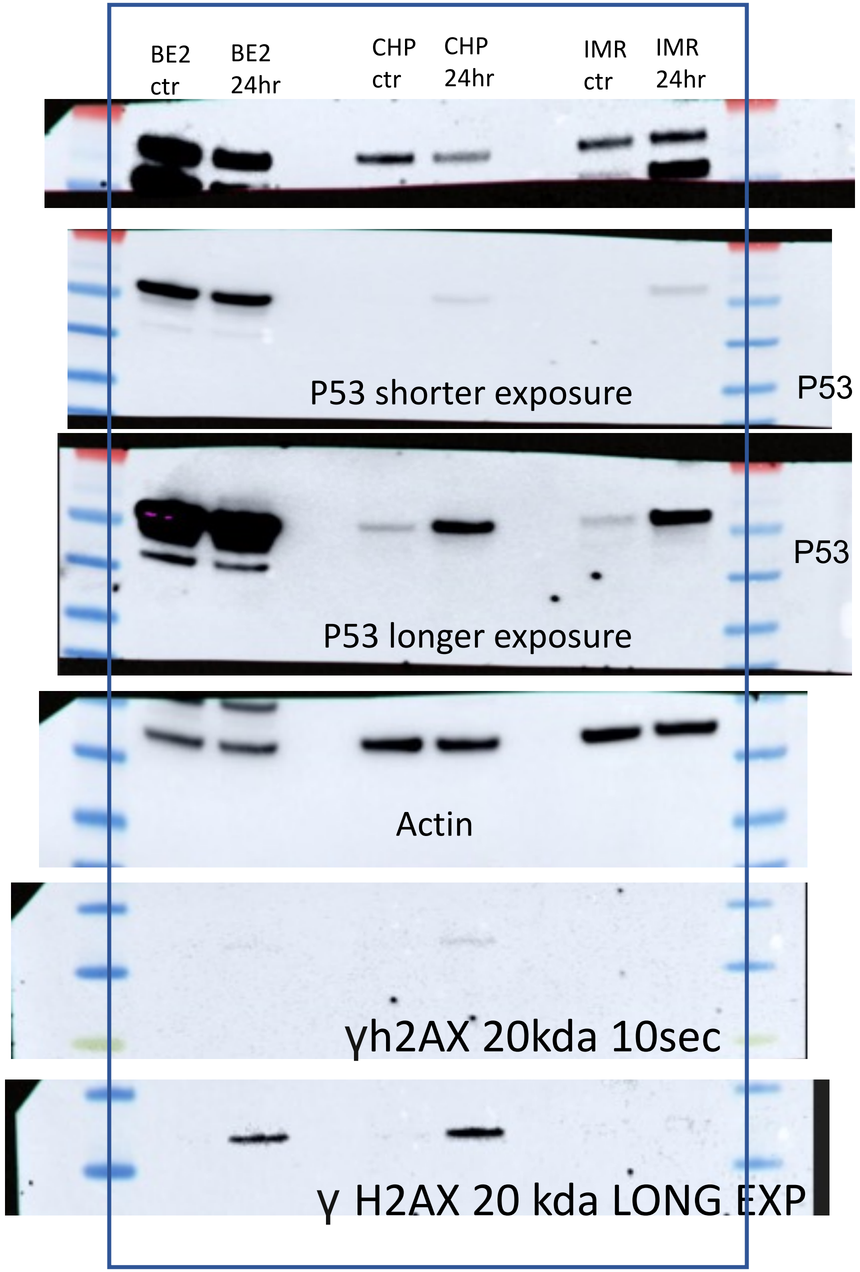

Supplementary Figure 2C

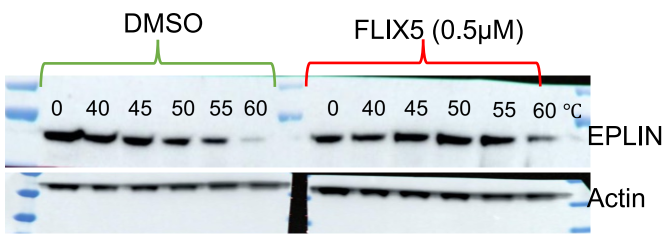

Supplementary Figure 5E

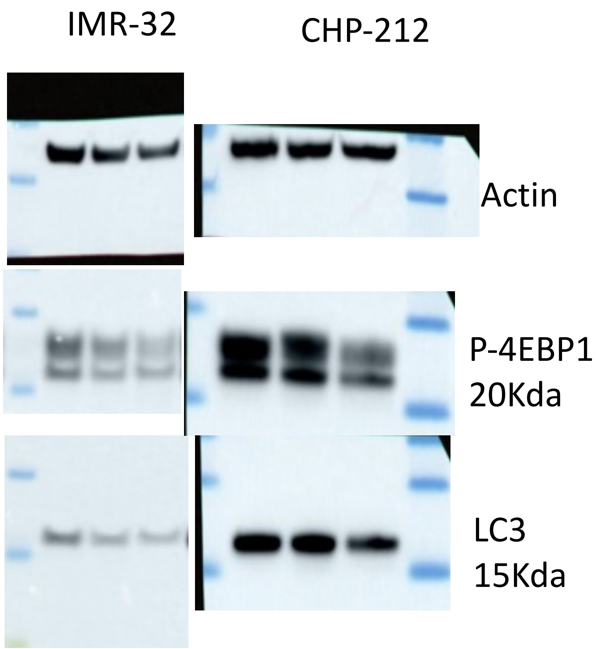

Supplementary Figure 7A

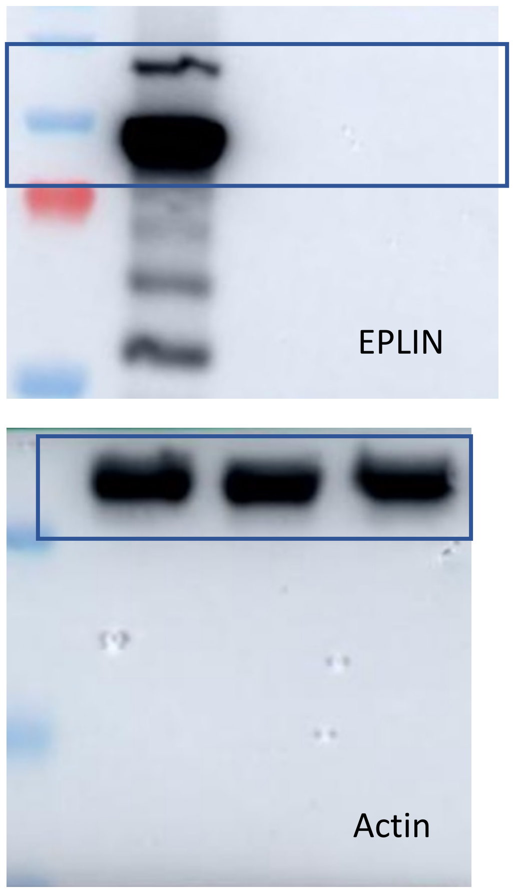

Supplement: Supplementary file 10 — Original WBs [file 41419_2025_7876_MOESM10_ESM.pdf]
